# The BHMT2/MAT1A/AHSG axis promotes M1 macrophage activation and exacerbates necrotizing enterocolitis

**DOI:** 10.1038/s41598-025-22915-1

**Published:** 2025-11-11

**Authors:** Wanyong Yue, Xingheng Lu, Wenmei Tian, Fei Zhang, Guowang Nong, Guangze Pan, Xueliang Zhang, Guodan Yu

**Affiliations:** Pediatric Surgery, The People’s Hospital of Wenshan Prefecture, No. 228, Kaihua Middle Road, Wenshan Prefecture, Wenshan, 663000 Yunnan China

**Keywords:** Necrotizing enterocolitis, AHSG, BHMT2, MAT1A, Macrophage polarization, Histone methylation, Epithelial cells, Cell biology, Molecular biology

## Abstract

Background Necrotizing enterocolitis (NEC) is a devastating intestinal disorder in premature infants, characterized by inflammation and tissue injury. Identifying key regulatory pathways contributing to NEC pathogenesis is essential for developing targeted therapeutic strategies. Methods Transcriptomic analysis of NEC and control samples identified a core regulatory module comprising AHSG, BHMT2, and MAT1A. Their expression and functional roles were investigated in human primary intestinal epithelial cells (HPIECs), a transwell co-culture system with THP-1 macrophages, and a mouse model of NEC. Molecular techniques, including RT-qPCR, Western blotting, ELISA, chromatin immunoprecipitation, and flow cytometry were employed to decipher the functional mechanism of this regulatory module. Results AHSG, BHMT2, and MAT1A were upregulated in NEC samples and LPS-stimulated HPIECs. BHMT2 and MAT1A regulated AHSG expression through S-adenosylmethionine production and histone methylation. In the co-culture system, silencing BHMT2, MAT1A, or AHSG in LPS-stimulated HPIECs attenuated M1 macrophage polarization, inflammatory cytokine production, and invasive capacity of THP-1 cells. Conversely, overexpressing these genes in HPIECs promoted M1 macrophage activation. In the NEC mouse model, targeting BHMT2, MAT1A, or AHSG alleviated intestinal tissue damage, inflammation, and M1 macrophage polarization. Conclusion The BHMT2/MAT1A/AHSG axis in intestinal epithelial cells orchestrates M1 macrophage activation and contributes to the exacerbation of NEC. Targeting this pathway may represent a potential therapeutic strategy for NEC management.

**Supplementary Information:**

The online version contains supplementary material available at 10.1038/s41598-025-22915-1.

## Introduction

Necrotizing enterocolitis (NEC) is a devastating gastrointestinal emergency that primarily affects premature infants, characterized by intestinal inflammation, tissue necrosis, and multi-organ dysfunction^1^. Despite advances in neonatal care, NEC remains a significant cause of morbidity and mortality, with an estimated incidence of 7% in infants weighing less than 1500 g^2^. The pathogenesis of NEC is multifactorial, involving an interplay between intestinal immaturity, bacterial colonization, inflammatory cascades, and ischemic injury^3^. Current treatment strategies for NEC are largely supportive, including bowel rest, broad-spectrum antibiotics, and surgical intervention in severe cases, highlighting the need for novel therapeutic approaches targeting the underlying molecular mechanisms^4^.

Emerging evidence suggests that the crosstalk between intestinal epithelial cells and macrophages plays a crucial role in the initiation and perpetuation of the inflammatory response in NEC^5,6^. Intestinal epithelial cells, which serve as the first line of defense against pathogens and noxious stimuli, can secrete various cytokines and chemokines that shape the inflammatory milieu and influence macrophage activation^7^. Conversely, activated macrophages release a plethora of inflammatory mediators, including reactive oxygen species, cytokines, and proteolytic enzymes, which can further disrupt the epithelial barrier and exacerbate tissue injury^8^. Dysregulated activation of macrophages, particularly the pro-inflammatory M1 phenotype, contributes to the exacerbation of tissue damage and the amplification of inflammatory signaling in NEC^9,10^. In contrast, the anti-inflammatory M2 macrophage phenotype has been shown to promote tissue repair and resolution of inflammation^11^. Understanding the regulatory pathways governing macrophage polarization and function in the context of NEC is pivotal for developing targeted interventions.

Alpha-2-HS-glycoprotein (AHSG), also known as fetuin-A, is a multifunctional glycoprotein involved in various biological processes, including inflammation and macrophage regulation^12^. Previous studies have demonstrated that AHSG can modulate macrophage polarization, promoting the M1 pro-inflammatory phenotype and inhibiting the anti-inflammatory M2 state^13,14^. However, the role of AHSG and its regulatory mechanisms in NEC pathogenesis remain largely unexplored.

In this study, we aimed to elucidate the molecular pathways governing AHSG expression and its functional implications in macrophage activation and NEC progression. Through transcriptomic analysis, we identified a core regulatory module involving AHSG, betaine–homocysteine S-methyltransferase 2 (BHMT2), and methionine adenosyltransferase 1 A (MAT1A). We investigated the interplay between this regulatory axis, epithelial cell-derived AHSG, and macrophage polarization using in vitro co-culture systems and an in vivo NEC mouse model. Our findings provide insights into the potential therapeutic targeting of this pathway for the management of NEC.

## Methods

### Public data retrieval

RNA-sequencing data for necrotizing enterocolitis (NEC) and control samples (GSE200929) were retrieved from the Gene Expression Omnibus (GEO) database. Differentially expressed genes (DEGs) were identified using the DESeq2 R package with an adjusted p-value < 0.05 and |log2(fold change)| > 1 as the cutoff criteria. The protein-protein interaction (PPI) network was constructed based on the protein pair information extracted from STRING database. Gene set enrichment analysis was performed using the gene sets catalogued by Kyoto Encyclopedia of Genes and Genomes (KEGG) database (www.kegg.jp/kegg/kegg1.html)^15^.

### Clinical sample collection

Control and NEC tissue samples were collected from patients at Ethics Committee of The People’s Hospital of Wenshan Prefecture (WYLS2023037) following institutional research ethics board approval, and all enrolled subjects provided the written informed consent. Samples were immediately snap-frozen in liquid nitrogen and stored at −80 °C until further analysis.

### Cell culture and treatment

Human primary intestinal epithelial cells (HPIECs, Cell Biologics Inc.) were cultured in Epithelial Cell Growth Medium (iXCells BioTechnologies). HPIECs were treated with 100 ng/mL lipopolysaccharide (LPS; Sigma-Aldrich) for 24 h to simulate inflammatory conditions. THP-1 cells (Procell) were cultivated in RPMI-1640 medium supplemented with 10% FBS (Procell). All the cells were maintained in a humidified atmosphere at 37 °C and 5% CO_2_.

### Co-culture experiment

A transwell co-culture system was established using 24-well Transwell plates with 0.4 μm pore size polycarbonate membrane inserts (Corning Costar). HPIECs were seeded in the lower chamber and allowed to form a confluent monolayer. The cells were then transfected with siRNAs or expression vectors as described above, and stimulated with 100 ng/mL LPS for 24 h. The human monocytic cell line THP-1 was seeded in the upper chamber inserts at a density of 1 × 10^5 cells/insert. After 48 h of co-culture, THP-1 cells were collected from the upper chamber and analyzed for macrophage polarization markers and functional assays as detailed below.

### Cellular transfection

The siRNAs used in this study were synthesized by MedChemExpress. HPIECs were transfected with 50 nM siRNAs targeting BHMT2, MAT1A, AHSG, or non-targeting control siRNA using Lipofectamine RNAiMAX (Invitrogen). For overexpression studies, HPIECs were transfected with expression vectors encoding BHMT2, MAT1A, AHSG, or empty vector control using Lipofectamine 3000 (Invitrogen). Cells were used for functional experiments 48 h after transfection.

### Real-time qPCR analysis

Total RNA was extracted using the RNeasy Mini Kit (Qiagen), and 2 µg of total RNA sample was used for cDNA synthesis by utilizing the iScript cDNA Synthesis Kit (Bio-Rad). Real-time qPCR was performed using gene-specific primers and SYBR Green Master Mix (Applied Biosystems) on a QuantStudio 6 Flex System (Applied Biosystems). Gene expression was quantified using the 2^^−ΔΔCt^ method and normalized to GAPDH.

### Western blotting

Protein lysates were prepared in RIPA buffer (Beyotime) on ice for 15-minute incubation. After quantifying the concentration using a protein BCA assay kit (Beyotime), 10 µg of denatured protein sample was separated by SDS-PAGE, and transferred to PVDF membranes. After blocking with 5% non-fat milk for 1 h, membranes were incubated with primary antibodies against BHMT2, MAT1A, AHSG, and GAPDH (Cell Signaling Technology) at 4 °C for 24 h, followed by the labeling with HRP-conjugated secondary antibody (Abcam). Proteins were detected using an enhanced ECL reagent (GE Healthcare) and imaged using a ChemiDoc system (Bio-Rad).

### Enzyme-linked immunosorbent assay (ELISA)

AHSG protein levels in culture media and tissue lysates were quantified using the human or mouse AHSG/Fetuin-A ELISA kit (R&D Systems) according to the manufacturer’s instructions. Inflammatory cytokine levels (TNF-α, IL-1β, and IL-6) were measured using respective ELISA kits (R&D Systems).

### CCK-8 cell growth assay

HPIECs were seeded in 96-well plates at a density of 3000 cells/well and treated as indicated. Cell growth was assessed at different time points using the CCK-8 assay kit (Dojindo Molecular Technologies). A total of 15 µL of CCK-8 reagent was added to each well at indicated time point for 4-hour incubation, and then the supernatant was discarded. The cells were dissolved in 150 µL DMSO at 37 °C for 10 min, and the optical density was measured at 570 nm.

### Transwell invasion assay

THP-1 cells were seeded in the upper chamber of Matrigel-coated Transwell inserts (Corning) at a density of 2.5 × 10^5^ cells per well in serum-free medium, while HPIECs were cultured in the lower chamber. After co-culture, cells that invaded through the Matrigel were fixed by 4% formaldehyde for 10 min, stained with 0.2% crystal violet (Beyotime), and counted under a light microscope.

### Apoptosis and macrophage surface marker analysis by flow cytometry

For apoptosis analysis, 1 × 10^6^ of HPIECs were stained with Annexin V-FITC and propidium iodide reagent for 15 min using the Annexin V Apoptosis Detection Kit (BD Biosciences). For macrophage polarization analysis, cells were stained with APC-conjugated anti-CD86 (M1 marker) and PE-conjugated anti-CD206 (M2 marker) antibodies (BioLegend) on ice for 15 min. The cell samples were analyzed using BD FACSCalibur flow cytometer (BD Biosciences).

### Animal model and in vivo treatment

All animal procedures were conducted in accordance with ARRIVE guidelines and relevant institutional guidelines and regulations. C57BL/6 mice were obtained from Charles River Laboratories Beijing. The NEC mouse model was established as previously described^16,17^. Briefly, premature newborn pups (C57BL/6 mice) were delivered by cesarean section at embryonic day 18 and exposed to the following stressors to induce NEC-like intestinal injury: single dose of intragastric LPS (2 mg/kg) 48 h after birth; Hypoxia (95% N2 and 5% O2) for 10 min twice daily; Hypothermia (placing pups on a 4 °C aluminum plate for 10 min twice daily); Hypertonic formula feeding (Similac 60, Ross Laboratories, 0.2 mL every 3 h). All experimental procedures involving live animals were approved by the Ethical Review Committee for Animal Experiments of Kunming Medical University (approval number: kmmu20241651) and were performed in strict accordance with institutional guidelines and national regulations for the care and use of laboratory animals. The control littermates were maintained with the dam and received no interventions. For lentiviral shRNA treatment, pups were randomized to receive intraperitoneal injections of lentiviruses encoding shRNAs targeting BHMT2 or MAT1A (1 × 10^7 transducing units in 30 µL PBS) or a non-targeting control shRNA on days 1 and 4 of life. For antibody neutralization studies, pups were randomized to receive intraperitoneal injections of anti-AHSG neutralizing antibody (R&D Systems, 100 µg in 30 µL PBS) or isotype control IgG on days 1 and 4 of life. For surgical procedures and tissue collection, mice were anesthetized with isoflurane (2–3% for induction, 1–2% for maintenance) delivered in oxygen. Pups were euthanized on day 7 by carbon dioxide (CO2) inhalation followed by cervical dislocation to ensure death. During the CO2 euthanasia, the flow rate was set to displace 20% of the chamber volume per minute to minimize distress. Intestinal tissues were collected for histological analysis, protein/RNA.

### Isolation of macrophages from intestinal tissue

Intestinal tissues were harvested from mice at day 7 after NEC induction. The tissues were opened longitudinally, washed in cold PBS to remove fecal contents, and cut into 1–2 cm pieces. The tissue pieces were incubated in HBSS containing 5 mM EDTA and 1 mM DTT at 37 °C for 20 min with gentle shaking to remove epithelial cells. The remaining tissue pieces were then digested in RPMI 1640 medium containing 10% FBS, 1 mg/mL collagenase IV (Sigma-Aldrich), and 30 U/mL DNase I (Roche) at 37 °C for 1 h with intermittent vigorous shaking. The digested tissues were passed through a 100 μm cell strainer (Falcon) to obtain a single-cell suspension. The cells were pelleted by centrifugation at 300 x g for 5 min, resuspended in MACS buffer (PBS with 0.5% BSA and 2 mM EDTA), and incubated with anti-CD11b magnetic beads (Miltenyi Biotec) for 15 min at 4 °C according to the manufacturer’s instructions. The CD11b^+^ macrophage population was isolated using LS columns on a MidiMACS separator (Miltenyi Biotec). The purity of the isolated macrophages was confirmed by flow cytometry analysis of CD11b expression.

### Histological analysis

Intestinal tissues were collected from mice, fixed in 4% paraformaldehyde for 24 h, and processed for paraffin embedding. Tissue Sect. (5 μm thick) were cut using a microtome and mounted on glass slides for hematoxylin and eosin (H&E) staining using the H&E Staining Kit (Solarbio, Beijing, China) according to the manufacturer’s instructions. Briefly, sections were deparaffinized in xylene, rehydrated through graded alcohols, stained with hematoxylin for 5 min, differentiated in acid alcohol, stained with eosin for 2 min, dehydrated, and mounted with neutral resin. For quantitative analysis of intestinal inflammation, a standardized histological scoring system (0–4 scale) was employed where score 0 indicated normal tissue with no inflammatory changes, score 1 represented mild inflammation with minimal inflammatory cell infiltration in the mucosal layer, score 2 denoted moderate inflammation with inflammatory cell infiltration in the mucosal layer and partial submucosal layer with mild villous structural damage, score 3 indicated severe inflammation with extensive inflammatory cell infiltration extending to the submucosal layer and significant villous structural destruction, and score 4 represented very severe inflammation with full-thickness inflammation, tissue necrosis, complete villous loss or ulcer formation. Histological scoring was performed by two independent researchers who were blinded to the experimental groups, and the final score for each sample was calculated as the average of the two independent assessments.

### Statistical analyses

Data are presented as mean ± SEM from at least three independent experiments. Statistical analyses were performed using GraphPad Prism software. Comparisons between two groups were evaluated by unpaired Student’s t-test, while multiple group comparisons were analyzed by one-way ANOVA followed by Tukey’s post-hoc test. A p-value < 0.05 was considered statistically significant.

## Results

### Identification of BHMT2/MAT1A/AHSG module in necrotizing enterocolitis (NEC)

**Fig. 1:**
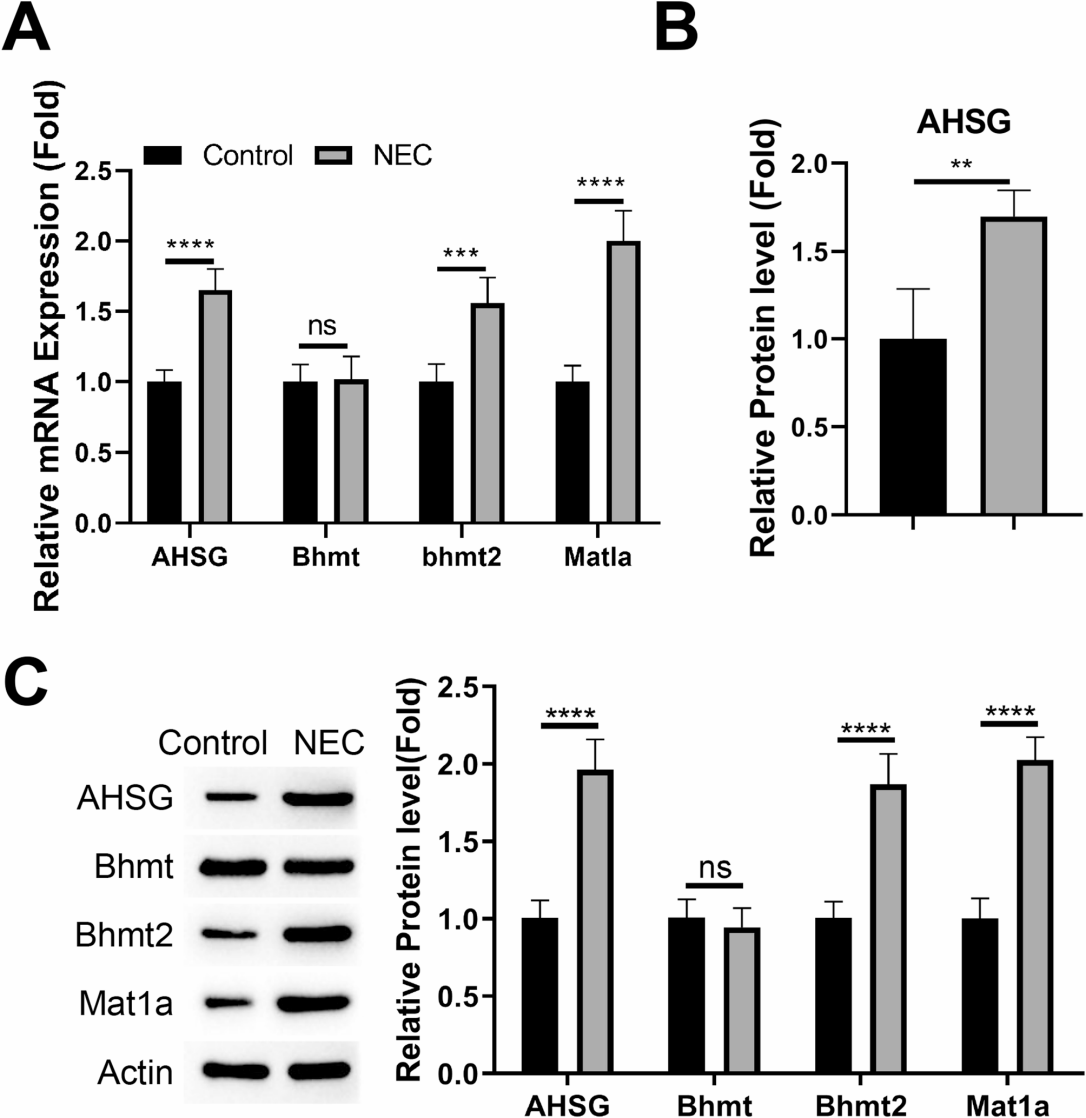
Identification of BHMT2/MAT1A/AHSG module in necrotizing enterocolitis (NEC). (**A**) Volcano plot showing differentially expressed genes (DEGs) in NEC vs. control samples. Red dots represent upregulated genes, and blue dots represent downregulated genes. (**B**) KEGG pathway enrichment analysis of DEGs using the KEGG database gene sets (www.kegg.jp/kegg/kegg1.html). (**C**) Protein-protein interaction (PPI) network of DEGs. The highlighted module indicates the core regulator module comprising AHSG, BHMT, BHMT2, and MAT1A. (**D**) Interaction scores of the core regulator module members.

To search for the underlying regulatory module in the progression of NEC, we retrieved the RNA-seq data (GSE200929, 3 control samples and 3 NEC samples) to profile the differentially expressed genes (DEGs). There were 380 DEGs in total, with 142 genes being upregulated and 238 genes downregulated (Fig. 1A). Enrichment analysis indicate that the major pathways affected by DEGs include humoral immune response, G-protein-coupled receptor binding and carbohydrate binding (Fig. 1B). We further created the protein-protein interaction (PPI) network of the DEGs, and identified a core regulator module with high interaction score (Fig. 1C,D). This module comprises 4 members: AHSG (Alpha 2-HS Glycoprotein, Fetuin A), BHMT (Betaine–Homocysteine S-Methyltransferase), BHMT2 and MAT1A (Methionine Adenosyltransferase 1 A). We focused on the BHMT2/MAT1A/AHSG axis as these three genes demonstrated the strongest interconnectivity within this module and represent a coherent metabolic pathway, although other downregulated genes may also play important regulatory roles that warrant future investigation.

**Fig. 2:**
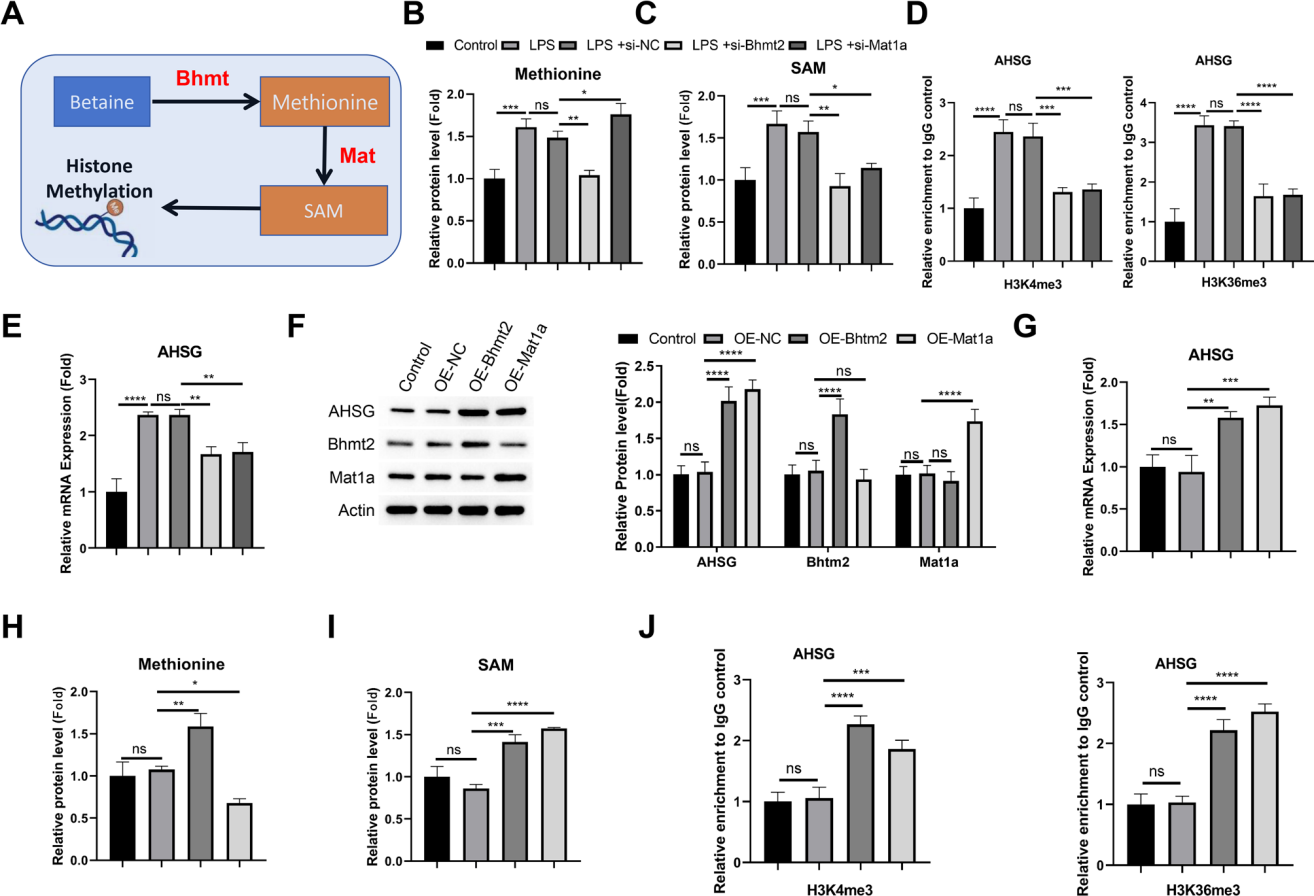
Validation of expression pattern of AHSG, BHMT, BHMT2, and MAT1A. (**A**) RT-qPCR analysis of AHSG, BHMT, BHMT2, and MAT1A mRNA levels in control and NEC tissue samples. (**B**) ELISA analysis of AHSG protein levels in control and NEC tissue samples. (**C**) Immunoblot analysis of AHSG, BHMT, BHMT2, and MAT1A protein levels in control and NEC tissue samples. *N* = 4 sample in each category. *p < 0.05;**p < 0.01;***p < 0.001; ****p < 0.0001.

To confirm these data, we collected control and NEC specimens for RT-qPCR verification. We found that AHSG, BHMT2 and MAT1A showed significant up-regulation in NEC samples (Fig. 2A), which is consistent with their trend in RNA-seq data. We further performed ELISA to analyze AHSG (Fetuin A) as it is a secreted glycoprotein involved in innate immunity^18,19^. In the NEC tissues, the protein level of AHSG was significantly elevated compared to the control (Fig. 2B). The increased expression of AHSG, BHMT2 and MAT1A was also confirmed at the protein level by immunoblotting (Fig. 2C).

### Increased expression of AHSG, BHMT2 and MAT1A in LPS-stimulated human primary intestinal epithelial cells (HPIECs)

**Fig. 3:**
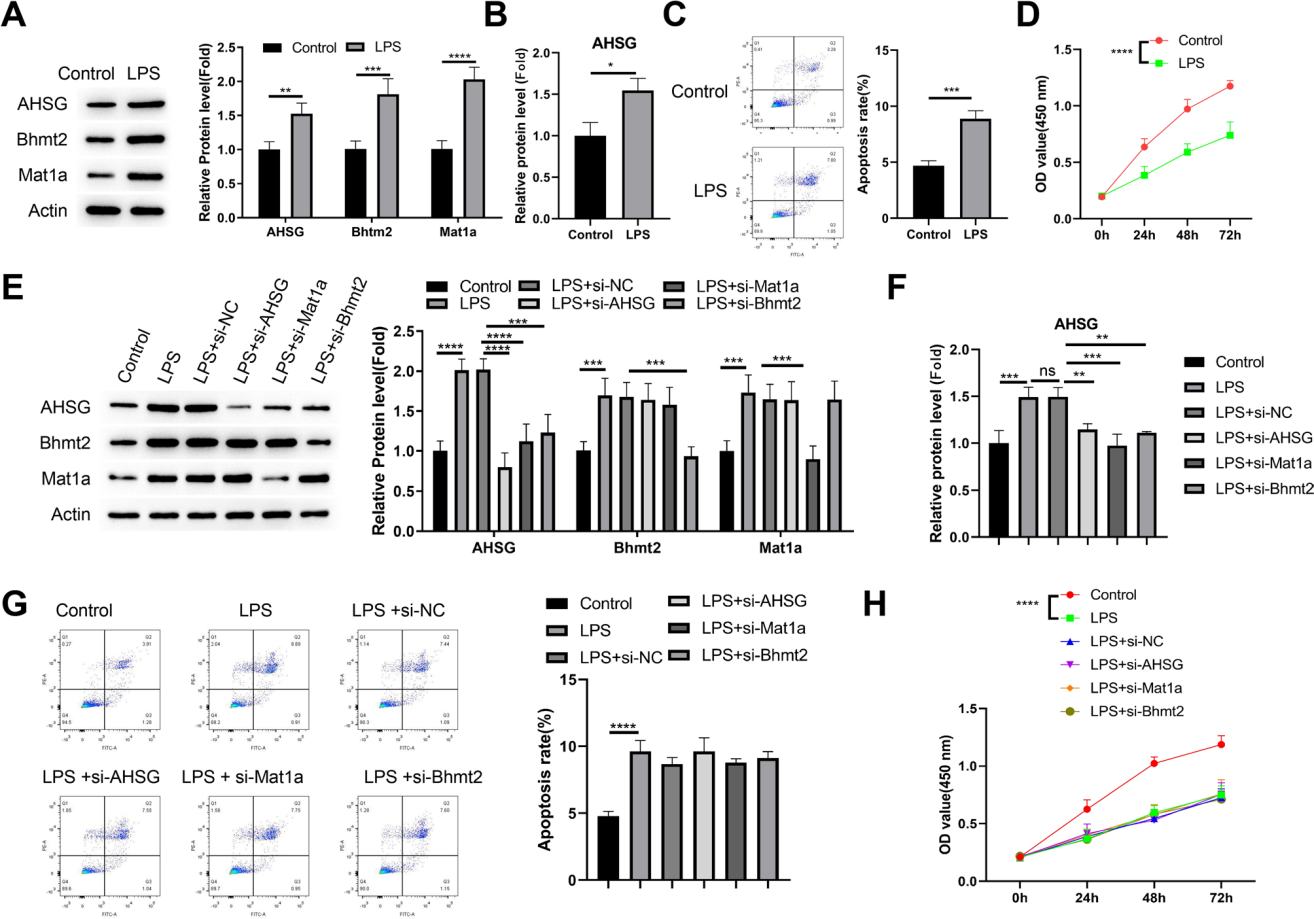
Increased expression of AHSG, BHMT2 and MAT1A in LPS-stimulated HPIECs. (**A**) RT-qPCR analysis of AHSG, BHMT2, and MAT1A mRNA levels in HPIECs with or without LPS treatment. (**B**) ELISA analysis of AHSG protein levels in culture media of HPIECs with or without LPS treatment. (**C**) Flow cytometry analysis of apoptotic cell death in HPIECs with or without LPS treatment. (**D**) Cell proliferation assay of HPIECs with or without LPS treatment. (**E**) Immunoblot analysis of AHSG, BHMT2, and MAT1A protein levels in HPIECs transfected with siRNAs and treated with LPS. (**F**) ELISA analysis of AHSG protein levels in culture media of HPIECs transfected with siRNAs and treated with LPS. (**G**) Flow cytometry analysis of apoptotic cell death in HPIECs transfected with siRNAs and treated with LPS. (**H**) Cell proliferation assay of HPIECs transfected with siRNAs and treated with LPS. *N* = 3 independent experiments. *p < 0.05;**p < 0.01;***p < 0.001; ****p < 0.0001.

To study the functional role of this regulatory module, we stimulated HPIECs with LPS to simulate the NEC inflammatory condition. We found that LPS treatment could upregulate the expressions of AHSG, BHMT2 and MAT1A (Fig. 3A). In addition, ELISA results showed that AHSG protein level in the culture medium was also increased after LPS stimulation (Fig. 3B). Flow cytometry analysis showed an increase of apoptotic cell death in HPIECs after LPS treatment (Fig. 3C), and LPS induction also suppressed the growth of HPIECs (Fig. 3D). To investigate whether the over-expression of AHSG, BHMT2 and MAT1A mediates LPS-induced cell death and growth arrest, we transfected HPIECs with control siRNA or the siRNA targeting each gene. Immunoblotting data demonstrated that each siRNA successfully reduced the expression of the target gene in LPS-stimulated HPIECs (Fig. 3E). Interestingly, we found that silencing BHMT2 or MAT1A also suppressed the expression of AHSG, which was confirmed by the ELISA analysis (Fig. 3F). Nevertheless, apoptosis and proliferation analysis showed that silencing these genes did not rescue the effect of LPS induction (Fig. 3G and H). These data suggest that the up-regulation of BHMT2/MAT1A/AHSG module may not directly cause detrimental effect on HPIECs.

### BHMT2 and MAT1A are required for AHSG up-regulation through SAM production and histone methylation

**Fig. 4:**
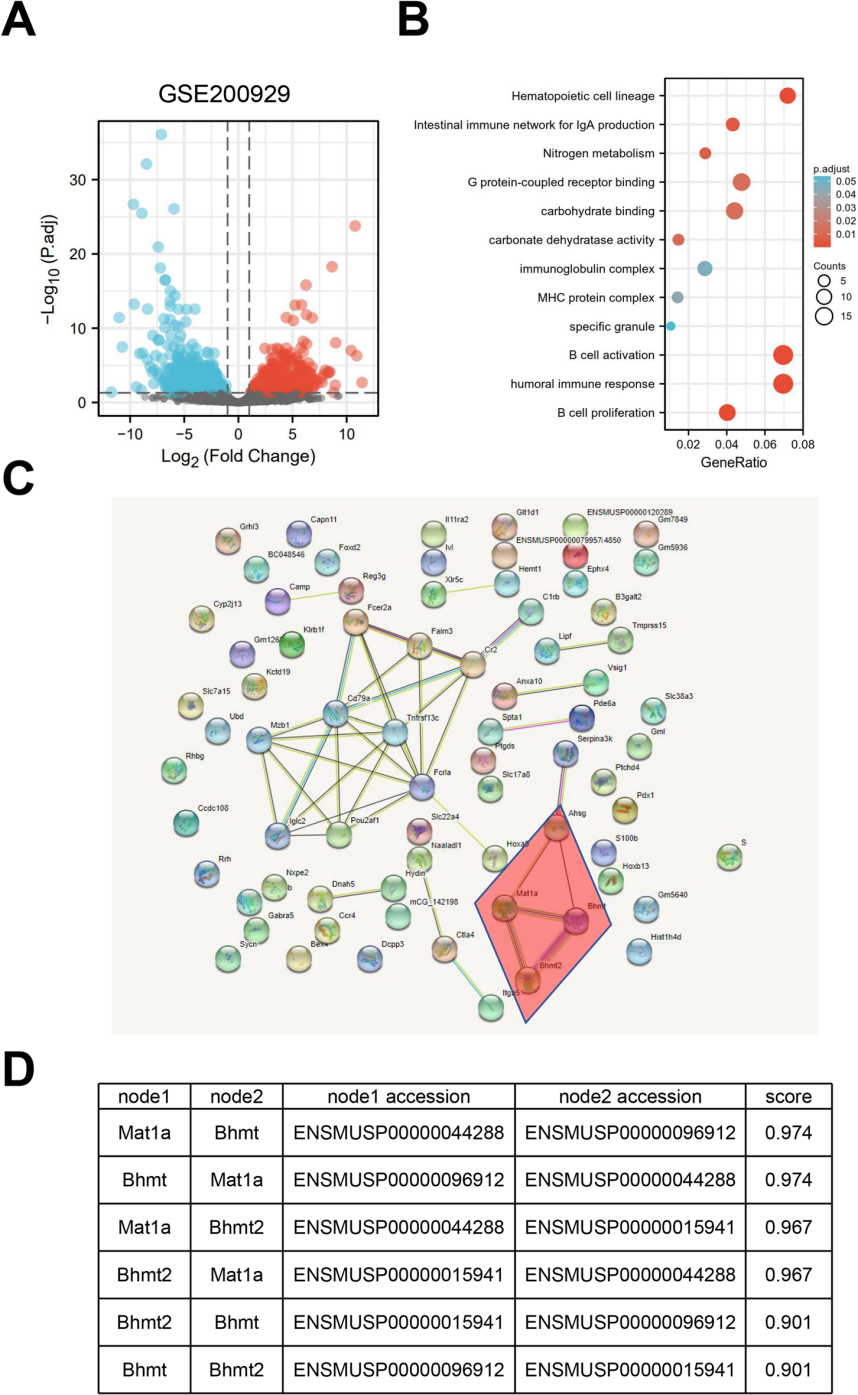
BHMT2 and MAT1A are required for AHSG up-regulation through SAM production and histone methylation. (**A**) Schematic of the two-step process for S-adenosylmethionine (SAM) generation involving BHMT2 and MAT1A. (**B**) Methionine levels in HPIECs transfected with siRNAs and treated with LPS. (**C**) SAM levels in HPIECs transfected with siRNAs and treated with LPS. (**D**) ChIP-qPCR analysis of H3K4me3 and H3K36me3 enrichment at the AHSG gene locus in HPIECs transfected with siRNAs and treated with LPS. (**E**) RT-qPCR analysis of AHSG mRNA levels in HPIECs transfected with siRNAs and treated with LPS. (**F**) Immunoblot analysis of AHSG, BHMT2, and MAT1A protein levels in HPIECs transfected with BHMT2 or MAT1A expression vector. (**G**) RT-qPCR analysis of AHSG mRNA levels in HPIECs transfected with BHMT2 or MAT1A expression vector. (**H**) Methionine levels in HPIECs transfected with with BHMT2 or MAT1A expression vector. (**I**) SAM levels in HPIECs transfected with BHMT2 or MAT1A expression vector. (**J**) ChIP-qPCR analysis of H3K4me3 and H3K36me3 enrichment at the AHSG gene locus in HPIECs transfected with BHMT2 or MAT1A expression vector. *N* = 3 independent experiments. *p < 0.05;**p < 0.01;***p < 0.001; ****p < 0.0001.

BHMT2 and MAT1A are involved in a two step process for the generation of S-adenosylmethionine (SAM) for DNA and histone methylation. BHMT2 function to add a methyl group to homocysteine from betaine to create methionine, and MAT1A moves the adenosyl group from ATP to methionine to generate SAM^20,21^ (Fig. 4A). Since we observed that silencing BHMT2 or MAT1A also suppressed the expression of AHSG, we wondered whether BHMT2 and MAT1A are responsible for AHSG up-regulation through histone methylation. To investigate this hypothesis, we measured methionine and SAM levels in LPS-stimulated HPIECs with or without gene silencing. We found that LPS induction caused an increase of methionine level, which is consistent with the up-regulation of BHMT2. Silencing BHMT2 reduced the methionine level under LPS stimulation, but MAT1A knockdown did not show this effect (Fig. 4B). LPS treatment also promoted the level of SAM in HPIECs, and both BHMT2 silencing and MAT1A knockdown could suppress the increase of SAM production after LPS stimulation (Fig. 4C). These data confirmed the role of BHMT2 and MAT1A in LPS-stimulated generation of SAM.

Next, we performed chromatin-immunoprecipitation (ChIP)-qPCR analysis using anti-H3K4Me3 antibody (histone mark for active gene promoter) and anti-H3K36Me3 antibody (histone mark for active transcribing gene body), with IgG isotype as the control. ChIP-qPCR results showed that both H3K4Me3 and H3K36Me3 levels were highly increased at AHSG gene locus after LPS induction, and BHMT2 or MAT1A knockdown could suppress the deposition of these active histone mark at AHSG gene (Fig. 4D). These changes were consistent with the qPCR results that silencing BHMT2 or MAT1A repressed the mRNA expression of AHSG gene in HPIECs (Fig. 4E).

To further corroborate the role of BHMT2 and MAT1A in AHSG gene upregulation, we constructed the expression vector for BHMT2 and MAT1A. The transfection of corresponding expression vector increased the protein levels of BHMT2 and MAT1A, and the overexpression of BHMT2 or MAT1A could also increase AHSG expression at both the protein and mRNA levels (Fig. 4F,G). BHMT2 over-expression promoted methionine production (Fig. 4H). Both BHMT2 and MAT1A over-expression increased SAM levels in HPIECs (Fig. 4I). ChIP-qPCR analysis showed that both BHMT2 and MAT1A over-expression could elevated the levels of H3K4Me3 and H3K36Me3 at AHSG gene locus (Fig. 4J). Together, these findings indicate that BHMT2 and MAT1A are required for AHSG up-regulation through SAM production and histone methylation in HPIECs.

### BHMT2/MAT1A/AHSG axis is required for M1 macrophage activation in the co-culture with LPS-stimulated HPIECs

**Fig. 5:**
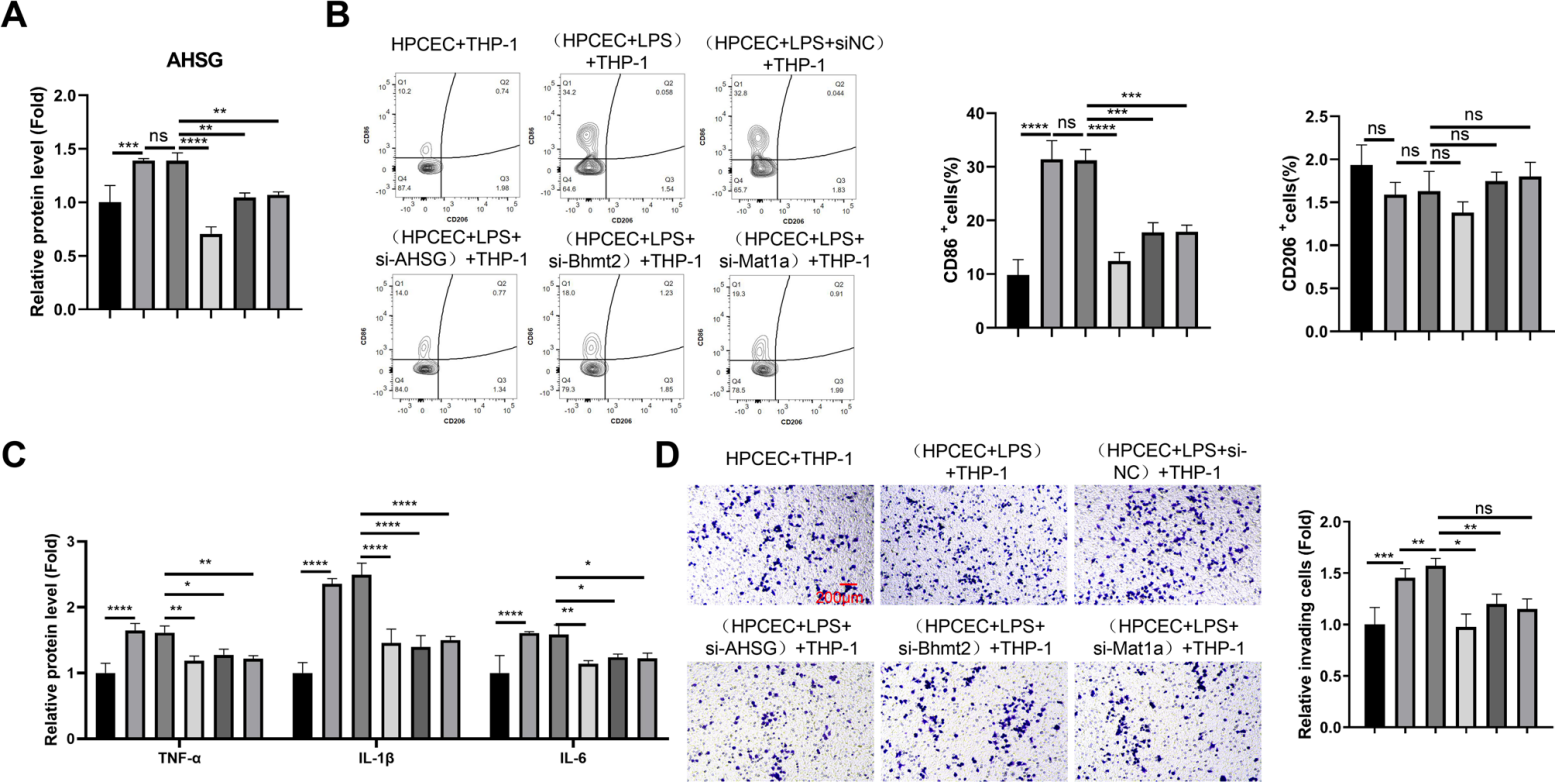
BHMT2/MAT1A/AHSG axis is required for M1 macrophage activation in the co-culture with LPS-stimulated HPIECs. (**A**) ELISA analysis of AHSG protein levels in culture media of HPIECs transfected with siRNAs and treated with LPS, used for co-culture with THP-1 cells. (**B**) Flow cytometry analysis of CD86 (M1 marker) and CD206 (M2 marker) levels in THP-1 cells after co-culture with different groups of HPIECs. (**C**) ELISA analysis of pro-inflammatory cytokine levels (TNF-α, IL-1β, IL-6) in THP-1 cells after co-culture with different groups of HPIECs. (**D**) Transwell invasion assay of THP-1 cells after co-culture with different groups of HPIECs. *N* = 3 independent experiments. *p < 0.05;**p < 0.01;***p < 0.001; ****p < 0.0001.

Pro-inflammatory activation of intestinal macrophage is a major contributor to the pathogenesis of NEC^22^. Since AHSG (Fetuin A) is implicated in macrophage polarization^23^, we next sought to clarify whether HPIEC-derived AHSG may impinge on the activation of macrophages. We established a transwell co-culture system between the human monocyte cell line THP-1 and different groups of HPIECs. HPIECs were transfected with a non-targeting siRNA (si-NC) or siRNAs targeting BHMT2, MAT1A, or AHSG, stimulated with LPS for 24 h, and then used for co-culture experiments. ELISA analysis showed that LPS treatment significantly increased AHSG protein levels in the culture medium, and silencing BHMT2, MAT1A, or AHSG in HPIECs reduced AHSG production (Fig. 5A). After 48 h of co-culture, we evaluated the polarization state of macrophages (M1-CD86, M2-CD206) using flow cytometry. Co-culture with LPS-stimulated HPIECs increased the percentage of CD86^+^ cells in THP-1 cells compared to co-culture with untreated HPIECs, and this effect was attenuated by silencing BHMT2, MAT1A, or AHSG in HPIECs (Fig. 5B). However, the percentages of CD206^+^ cells were comparable among different groups. ELISA analysis of pro-inflammatory cytokines (TNF-α, IL-1β and IL-6) in THP-1 cells also indicated inflammatory activation when co-cultured with LPS-stimulated HPIECs (Fig. 5C). Transwell invasion assays further showed an augmented invasive capacity of THP-1 cells in the LPS-stimulated HPIEC group (Fig. 5D). These effects were suppressed by silencing BHMT2, MAT1A, or AHSG in HPIECs. Collectively, our data indicate that the BHMT2/MAT1A/AHSG axis is required for M1 macrophage activation in co-culture with LPS-stimulated HPIECs.

### BHMT2/MAT1A/AHSG overexpression in HPIECs boosts M1 macrophage polarization

**Fig. 6:**
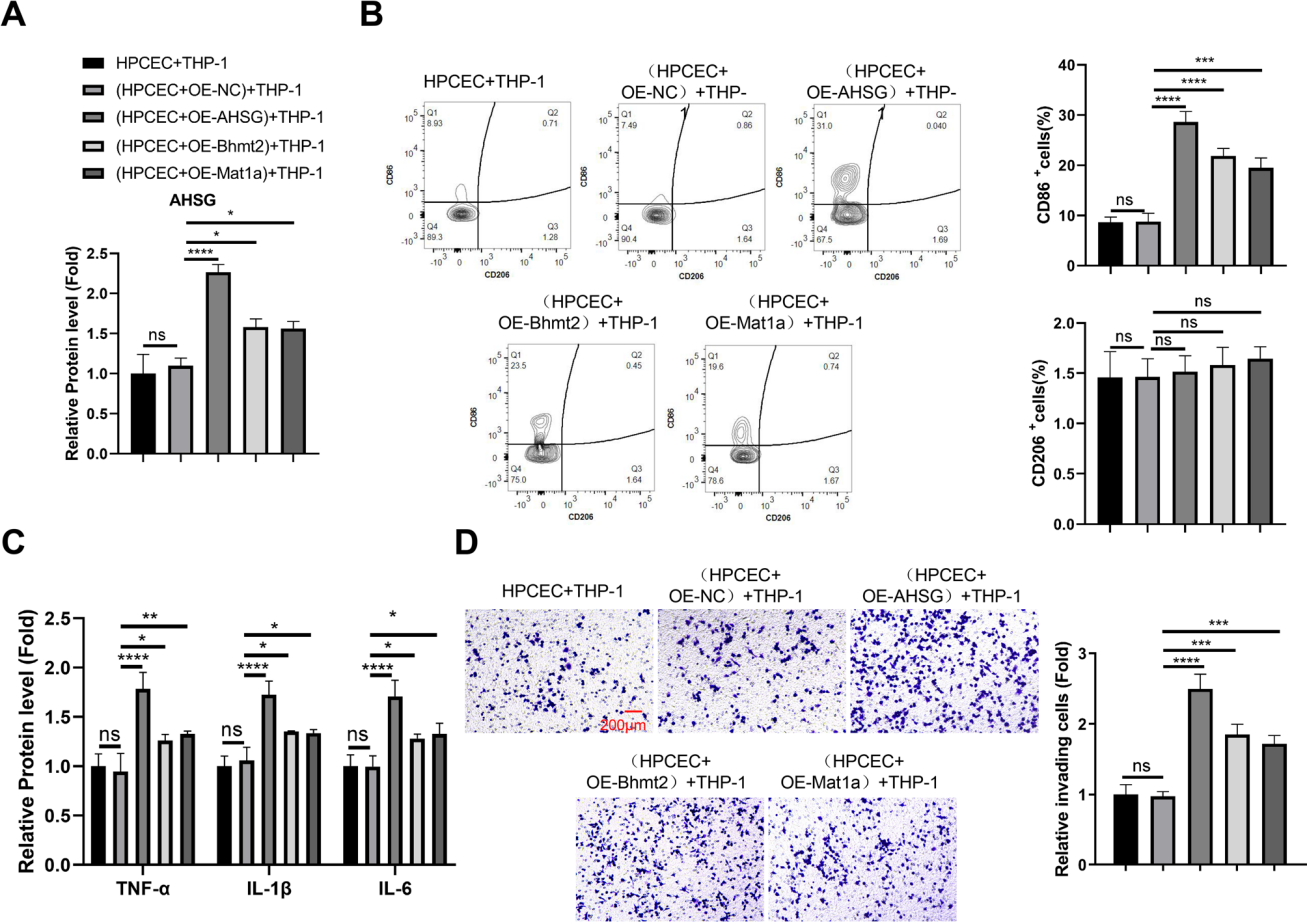
BHMT2/MAT1A/AHSG overexpression in HPIECs boosts M1 macrophage polarization. (**A**) ELISA analysis of AHSG protein levels in culture media of HPIECs transfected with expression vectors, used for co-culture with THP-1 cells. (**B**) Flow cytometry analysis of CD86 and CD206 expression in THP-1 cells after co-culture with HPIECs transfected with expression vectors. (**C**) ELISA analysis of pro-inflammatory cytokine levels (TNF-α, IL-1β, IL-6) in THP-1 cells after co-culture with HPIECs transfected with expression vectors. (**D**) Transwell invasion assay of THP-1 cells after co-culture with HPIECs transfected with expression vectors. *N* = 3 independent experiments. *p < 0.05;**p < 0.01;***p < 0.001; ****p < 0.0001.

We further overexpressed BHMT2, MAT1A and AHSG in HPIECs and used these cells in the co-culture with THP-1. As expected, the forced expression of BHMT2, MAT1A and AHSG increased the protein levels of AHSG in the culture medium (Fig. 6A). BHMT2, MAT1A and AHSG overexpression in HPIECs increased the percentages of CD86^+^ population in THP−1 cells (Fig. 6B), and promoted the production of inflammatory cytokines (Fig. 6C). The invasive capacity of THP−1 cells were also significantly augmented after the co-culture with BHMT2, MAT1A or AHSG-overexpressing HPIECs (Fig. 6D). Of note, HPIECs with AHSG overexpression showed the strongest effect on THP-1 cells, possibly due to the highest level of AHSG protein present in the culture medium.

### Targeting BHMT2/MAT1A and neutralizing AHSG protein alleviate NEC in a mouse model

**Fig. 7:**
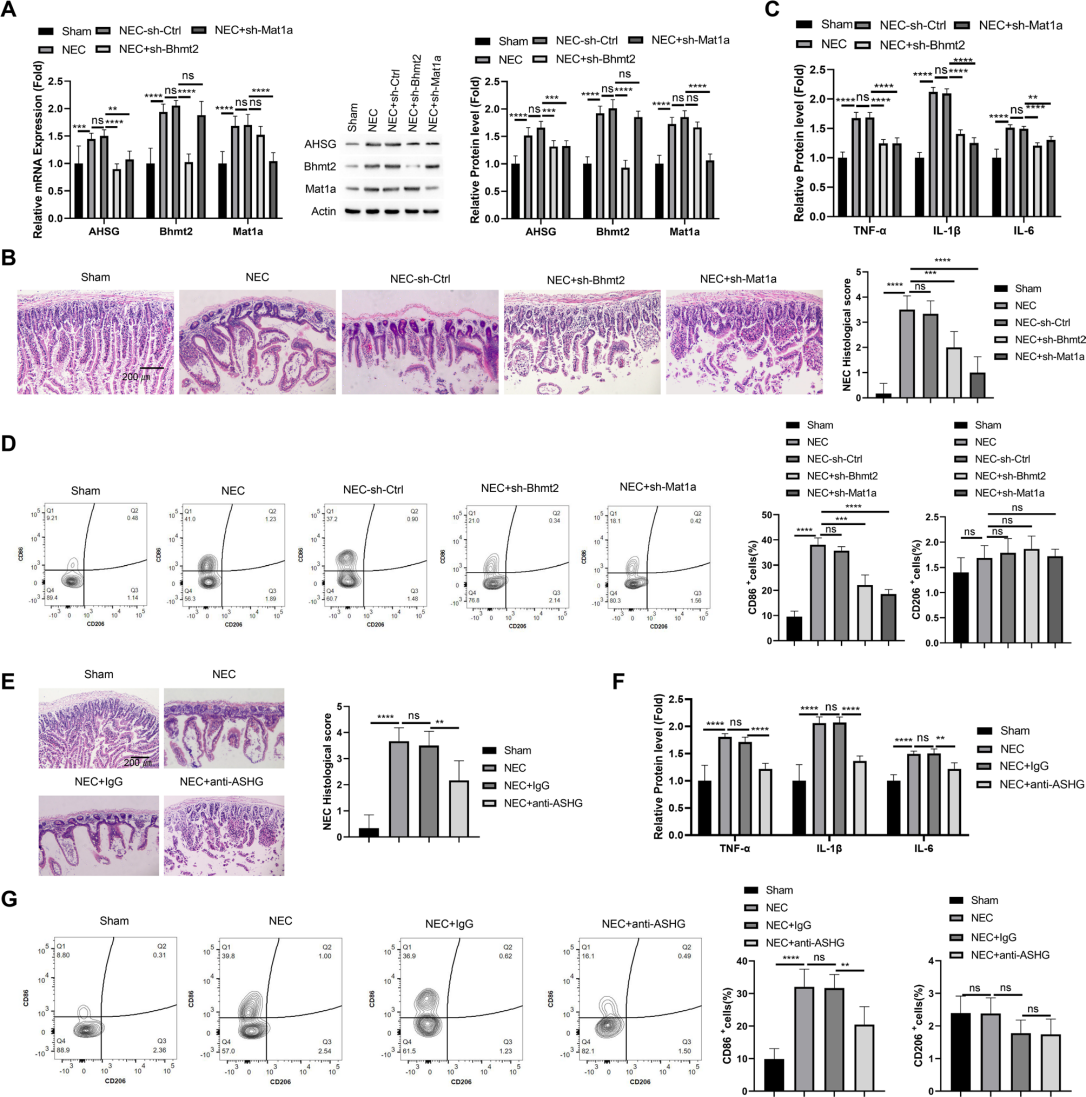
Targeting BHMT2/MAT1A and neutralizing AHSG protein alleviate NEC in a mouse model. (**A**) RT-qPCR and Western blot analysis of BHMT2, MAT1A, and AHSG mRNA and protein levels in intestinal tissues of NEC mouse model treated with control, BHMT2, or MAT1A shRNA lentiviruses. (**B**) Histological analysis of intestinal tissues of NEC mouse model treated with control, BHMT2, or MAT1A shRNA lentiviruses. (**C**) ELISA analysis of pro-inflammatory cytokine levels (TNF-α, IL-1β, IL-6) in intestinal tissues of NEC mouse model treated with control, BHMT2, or MAT1A shRNA lentiviruses. (**D**) Flow cytometry analysis of CD86 and CD206-expressing macrophages isolated from intestinal tissues of NEC mouse model treated with control, BHMT2, or MAT1A shRNA lentiviruses. (**E**) Histological analysis of intestinal tissues in NEC mouse model treated with IgG control or anti-AHSG neutralizing antibody. (**F**) ELISA analysis of pro-inflammatory cytokine levels (TNF-α, IL-1β, IL-6) in intestinal tissues of NEC mouse model treated with IgG control or anti-AHSG neutralizing antibody. (**G**) Flow cytometry analysis of CD86 and CD206-expressing macrophages isolated from intestinal tissues of NEC mouse model treated with IgG control or anti-AHSG neutralizing antibody. *N* = 6 animals per group. *p < 0.05;**p < 0.01;***p < 0.001; ****p < 0.0001.

To verify the role of BHMT2/MAT1A/AHSG axis in NEC progression, we established a mouse model and administrated lentivrius carrying control, BHMT2 or MAT1A shRNA. The introduction of BHMT2 and MAT1A shRNA suppressed the upregulation of both BHMT2 and MAT1A mRNA and protein levels in the intestinal tissues of NEC model, respectively. The expression of AHSG was also reduced (Fig. 7A). Histological analysis demonstrated that downregulating BHMT2 or MAT1A could protect against tissue damages in NEC model (Fig. 7B), as revealed by the histological staining and scores. The beneficial effect was accompanied by the reduction of inflammatory cytokines (TNF-α, IL-1β and IL-6) (Fig. 7C) and attenuated M1 polarization of macrophages in intestinal tissues (Fig. 7D). We further administrated anti-AHSG antibody or IgG isotype in the NEC model, and also found that AHSG neutralization could protect against intestinal tissue damages (Fig. 7E). AHSG neutralization was also able to suppress the production of inflammatory cytokines (Fig. 7F) and prevent M1 polarization of macrophages in intestinal tissues (Fig. 7G). Altogether, these data suggest that targeting BHMT2/MAT1A/AHSG axis may inhibit the exacerbation of NEC.

## Discussion

The findings of this study unravel a previously unrecognized regulatory axis involving AHSG, BHMT2, and MAT1A that orchestrates macrophage polarization and contributes to the exacerbation of necrotizing enterocolitis. Our results highlight the pivotal role of this pathway in modulating the crosstalk between intestinal epithelial cells and macrophages, a crucial determinant of NEC pathogenesis. Notably, our data suggest that AHSG, as the terminal effector of this axis, represents an attractive therapeutic target given its central role in driving macrophage polarization and its secreted nature that makes it accessible for intervention. Neutralizing this protein can be a promising therapeutic approach for mitigating inflammation and tissue injury in NEC. These insights provide a mechanistic framework for developing targeted therapeutic strategies aimed at addressing the inflammatory cascade in NEC.

The multifunctional glycoprotein AHSG (fetuin-A) has been implicated in various pathophysiological conditions, including inflammation, autoimmune disorders, and metabolic diseases^24^. Its role in macrophage polarization has been extensively studied, albeit with conflicting reports. Several studies have demonstrated that AHSG promotes the pro-inflammatory M1 macrophage phenotype, upregulating the expression of cytokines such as TNF-α, IL-1β, and IL-6^13,25^. In addition, the deficiency of AHSG was reported to protect against experimental autoimmune encephalomyelitis in the mouse model by dampening innate immunity^26^. Conversely, other reports suggest that AHSG induces an anti-inflammatory M2 macrophage state, characterized by increased production of IL-10 and decreased expression of inflammatory mediators^27,28^. These discrepancies may arise from the context-dependent effects of AHSG, influenced by factors such as tissue microenvironment, disease stage, and the presence of other regulatory factors. Our findings, however, indicate that AHSG derived from intestinal epithelial cells potentiates M1 macrophage activation and exacerbates inflammation in the context of NEC.

BHMT2 and MAT1A are key enzymes involved in the synthesis of S-adenosylmethionine (SAM), a universal methyl donor for various cellular processes, including DNA and histone methylation^29^. Dysregulation of BHMT2 and MAT1A has been implicated in various pathological conditions, such as liver diseases and cancer^30,31^. Previous studies have shown that BHMT2 and MAT1A can modulate gene expression through histone methylation, impacting cellular processes like cell proliferation, differentiation, and apoptosis^32,33^. Our findings extend this regulatory mechanism to the control of AHSG expression, demonstrating that BHMT2 and MAT1A are required for the upregulation of AHSG in intestinal epithelial cells through SAM-mediated histone methylation at the AHSG gene locus.

The detrimental role of M1 macrophages in the pathogenesis of inflammatory bowel diseases has been extensively documented. Excessive M1 macrophage activation leads to the overproduction of a plethora of pro-inflammatory cytokines, such as TNF-α, IL-1β, IL-6, and IL-12, as well as reactive oxygen species (ROS) and proteolytic enzymes^34,35^. These inflammatory mediators disrupt the intestinal epithelial barrier, promoting tight junction disruption, enterocyte apoptosis, and increased intestinal permeability^36^. Consequently, this facilitates bacterial translocation across the compromised epithelial barrier, triggering further inflammation and tissue injury in a self-perpetuating cycle^37^. Additionally, M1 macrophages have been shown to impair intestinal stem cell function and inhibit mucosal healing through the secretion of pro-inflammatory cytokines and exosomes^38,39^. This impairment of epithelial regeneration and tissue repair exacerbates the progression of NEC. Furthermore, M1 macrophages contribute to the development of NEC through their effects on other cellular components of the intestinal microenvironment, such as gut microbiota and intestinal epithelial cells^39,40^. Moreover, M1 macrophages can promote the recruitment and activation of neutrophils, amplifying the inflammatory response and exacerbating tissue damage through the release of granule enzymes and ROS^41,42^. Our findings corroborate these previous studies, demonstrating that epithelial cell-derived AHSG potentiates M1 macrophage polarization, inflammatory cytokine production, and invasive capacity, thereby contributing to the exacerbation of inflammation in NEC. Specifically, we observed an increased percentage of CD86^+^ (M1 marker) cells and elevated levels of pro-inflammatory cytokines (TNF-α, IL-1β, and IL-6) in THP-1 macrophages co-cultured with LPS-stimulated epithelial cells. Notably, these effects were attenuated by silencing BHMT2, MAT1A, or AHSG in epithelial cells, highlighting the pivotal role of this regulatory axis in driving M1 macrophage activation and inflammation. Importantly, our data suggest that the BHMT2/MAT1A/AHSG pathway functions primarily as a paracrine signaling mechanism, where epithelial cells secrete AHSG to modulate macrophage behavior rather than directly affecting epithelial cell survival. This explains why silencing these genes did not rescue LPS-induced epithelial cell damage in vitro, while anti-AHSG antibody treatment effectively ameliorated NEC in our animal model by interrupting the epithelial-macrophage inflammatory crosstalk.

While our study provides novel insights into the regulatory mechanisms underlying macrophage activation and NEC progression, there are certain limitations that warrant further investigation. First, the in vitro co-culture system, while informative, may not fully recapitulate the complex cellular interactions and microenvironmental cues present in the intestinal tissue. Future studies using more physiologically relevant models, such as organoid co-cultures or ex vivo intestinal tissue explants^43^, could further validate and expand upon our findings. Additionally, the specific mechanisms by which AHSG promotes M1 macrophage polarization, including potential receptor-mediated signaling pathways or epigenetic modifications, remain to be elucidated. Furthermore, while our study focused on macrophages as a critical immune cell population in NEC pathogenesis, we acknowledge that macrophage depletion studies would provide more definitive evidence for the causal role of the BHMT2/MAT1A/AHSG axis in macrophage-mediated intestinal inflammation. However, we cannot exclude that other immune cell components, including neutrophils, T cells, dendritic cells, and intestinal epithelial cells, may also be affected by the same BHMT2/MAT1A/AHSG axis and contribute to the overall inflammatory response in NEC. The complex intestinal immune microenvironment involves intricate crosstalk between multiple cell types, and the impact of this metabolic axis on the broader immune landscape requires comprehensive characterization. Despite these limitations, our study highlights the potential therapeutic value of targeting the BHMT2/MAT1A/AHSG axis in the management of NEC. Pharmacological inhibition of BHMT2 or MAT1A, or neutralization of AHSG using specific antibodies, may represent promising strategies to mitigate excessive inflammation and tissue injury. Moreover, modulating macrophage polarization through alternative approaches, such as targeted delivery of immunomodulatory agents or cell-based therapies^44,45^, could synergize with the inhibition of this regulatory pathway, offering a multifaceted approach to NEC treatment.

## Conclusion

In conclusion, our findings elucidate a novel regulatory pathway involving BHMT2, MAT1A, and AHSG that promotes M1 macrophage activation and exacerbates NEC progression. Targeting this axis may pave the way for the development of innovative therapeutic strategies aimed at mitigating inflammation and promoting intestinal mucosal healing in this devastating neonatal disorder.

## Supplementary Information

Below is the link to the electronic supplementary material.

Supplementary Material 1

## Data Availability

The data generated in this study are available upon request to the corresponding author.
