# Supplementary material for: The BHMT2/MAT1A/AHSG axis promotes M1 macrophage activation and exacerbates necrotizing enterocolitis
Source: Sci Rep. 2025 Nov 11;15:39458. doi: 10.1038/s41598-025-22915-1 (PMC12606173; doi:10.1038/s41598-025-22915-1)
Supplement: Supplementary file 1 — Supplementary Material 1 [file 41598_2025_22915_MOESM1_ESM.docx]

Figure 2C


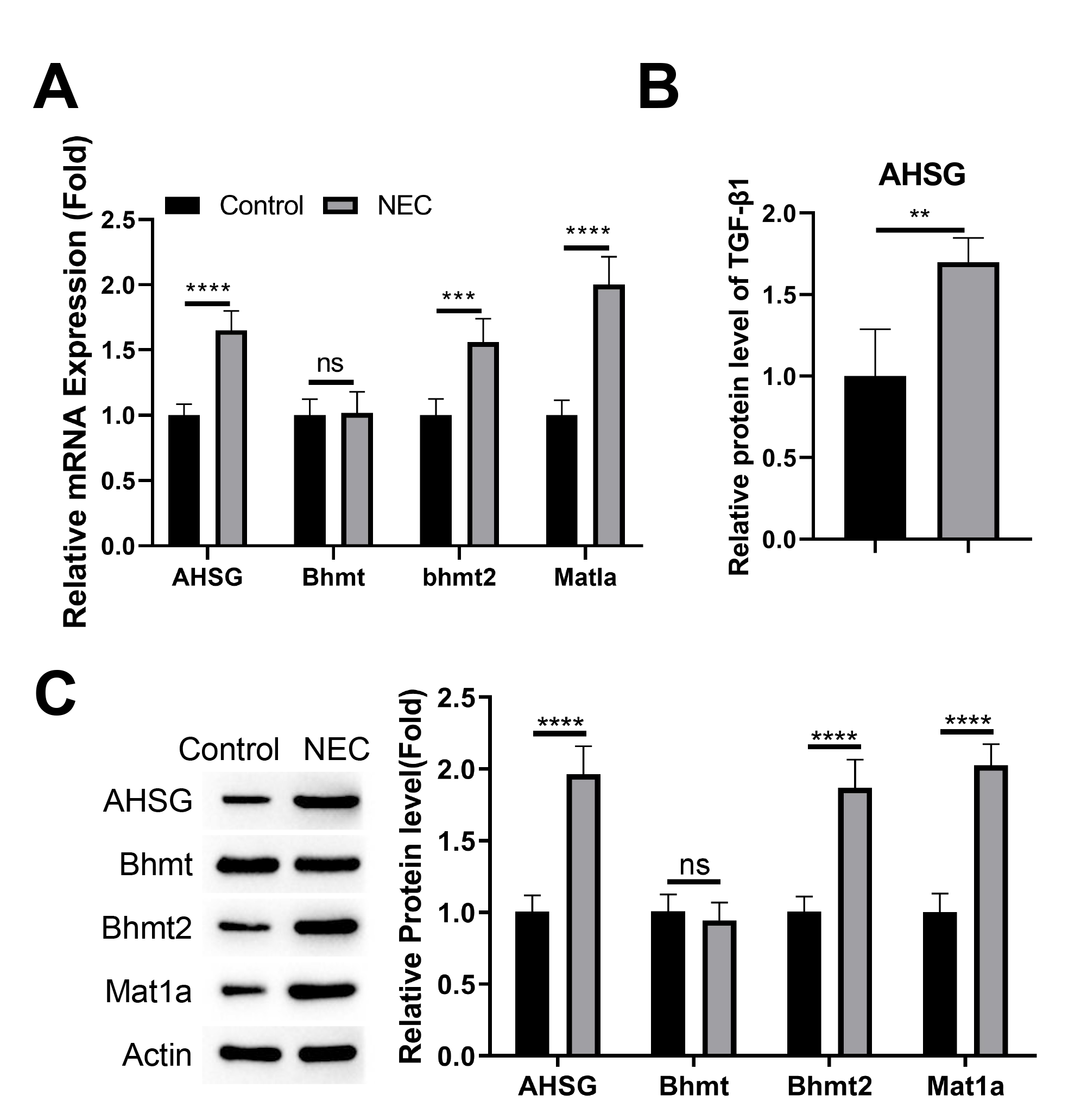


Actin

Membrane with ladder

Exposed signal


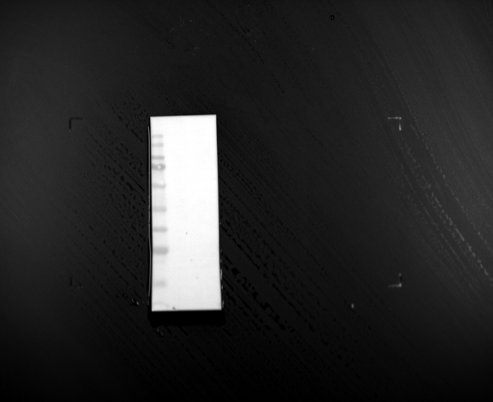

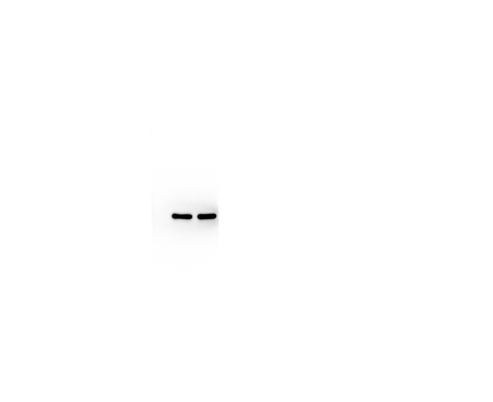

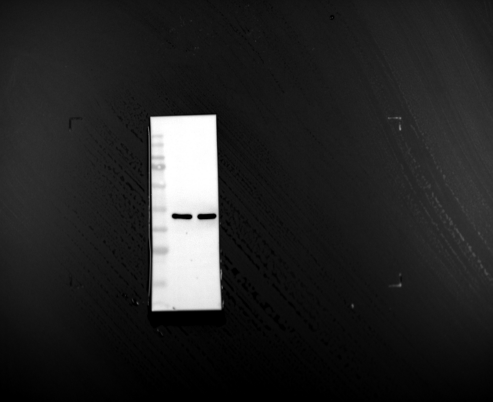


Molecular weight: 45 kDa

Merge

AHSG

Membrane with ladder

Exposed signal


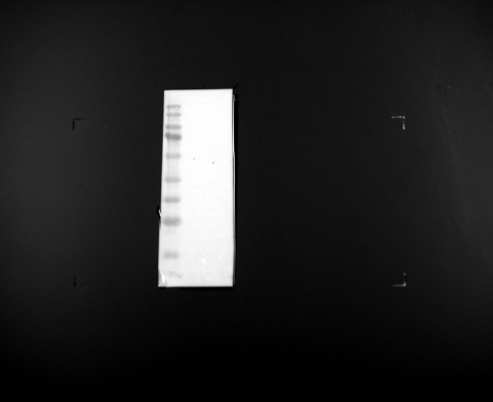

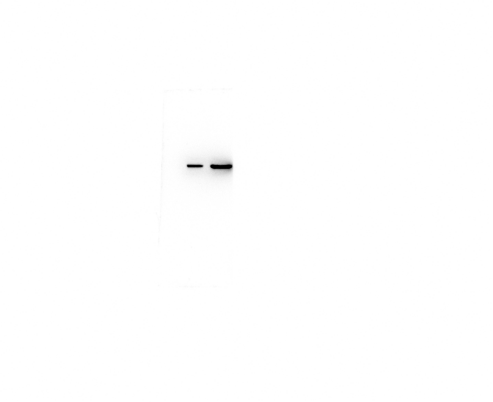

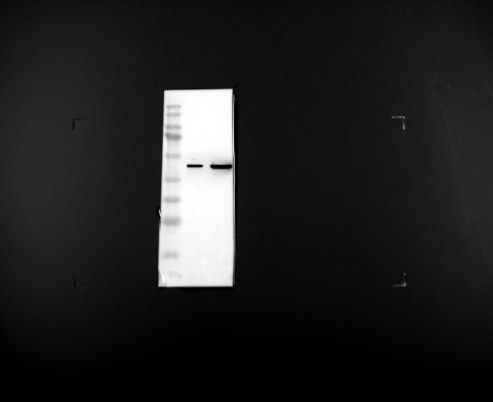


Molecular weight:55 kDa

Merge

Bhmt

Membrane with ladder

Exposed signal


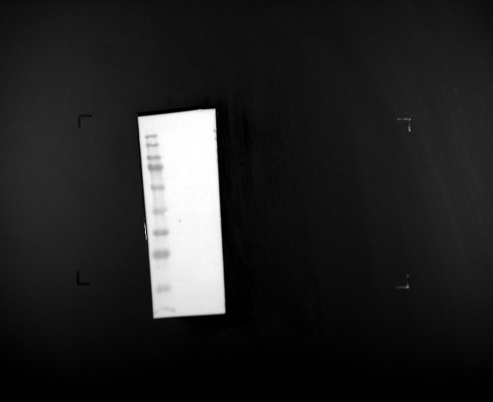

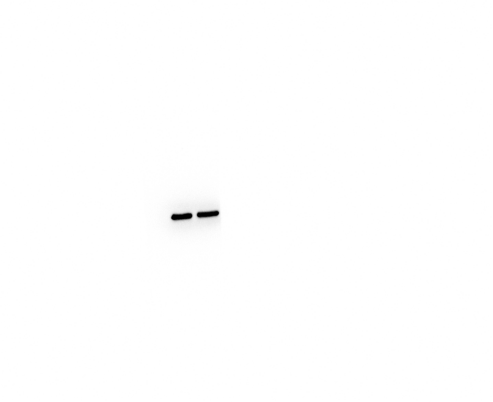

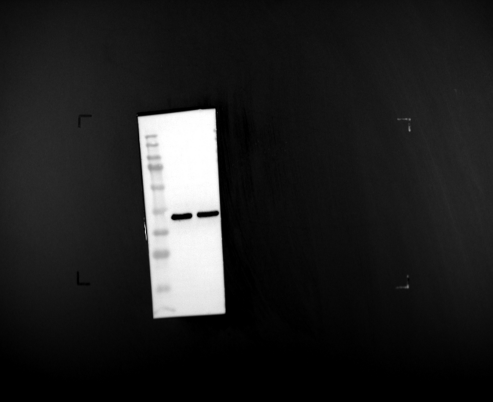


Molecular weight: 45 kDa

Merge

Bhmt2

Membrane with ladder

Exposed signal


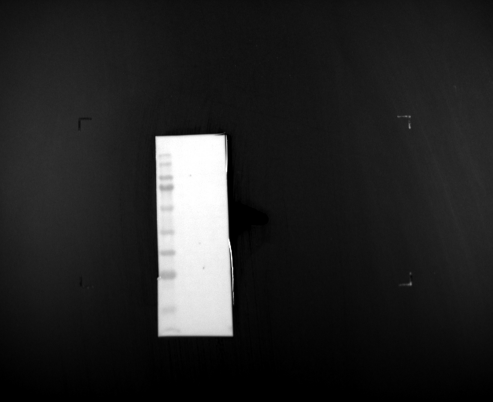

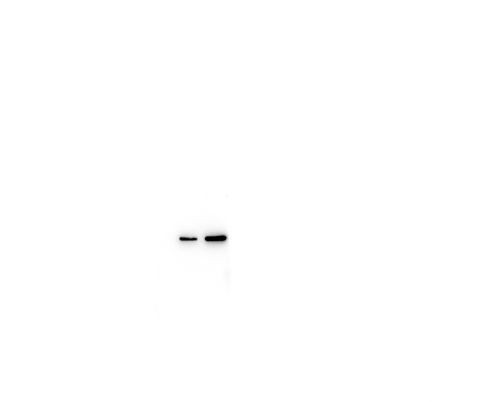

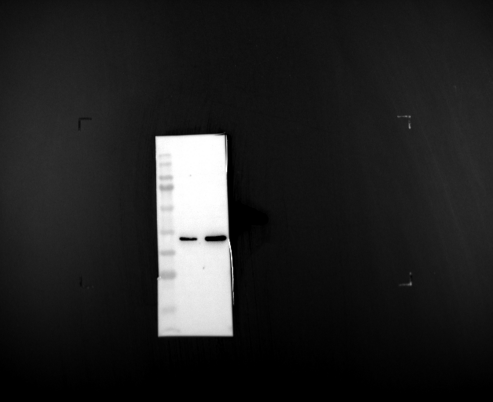


Molecular weight: 40-45 kDa

Merge

Mat1a

Membrane with ladder

Exposed signal


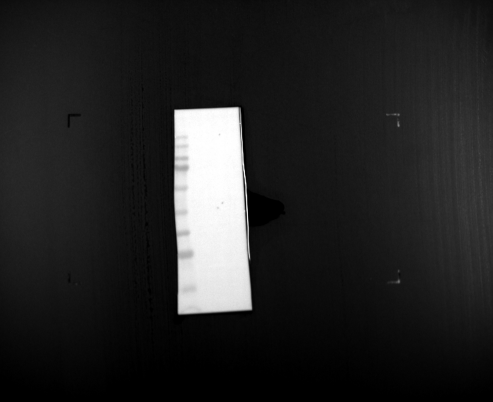

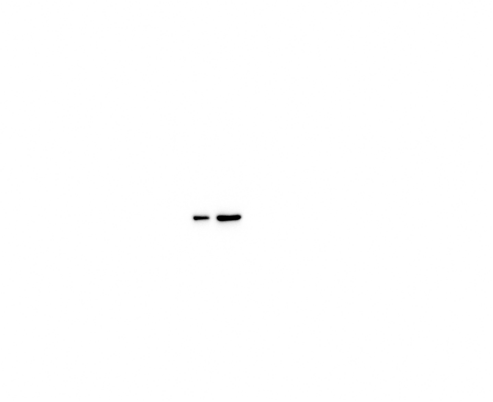

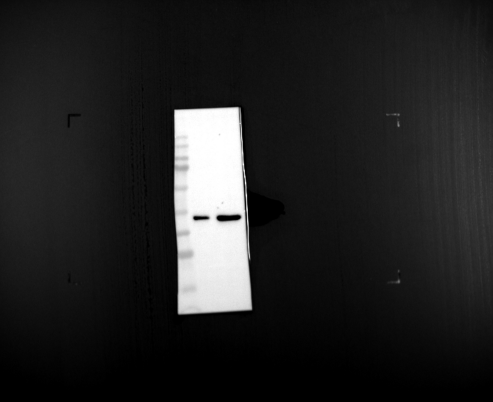


Molecular weight: 44 kDa

Merge

Figure 3A


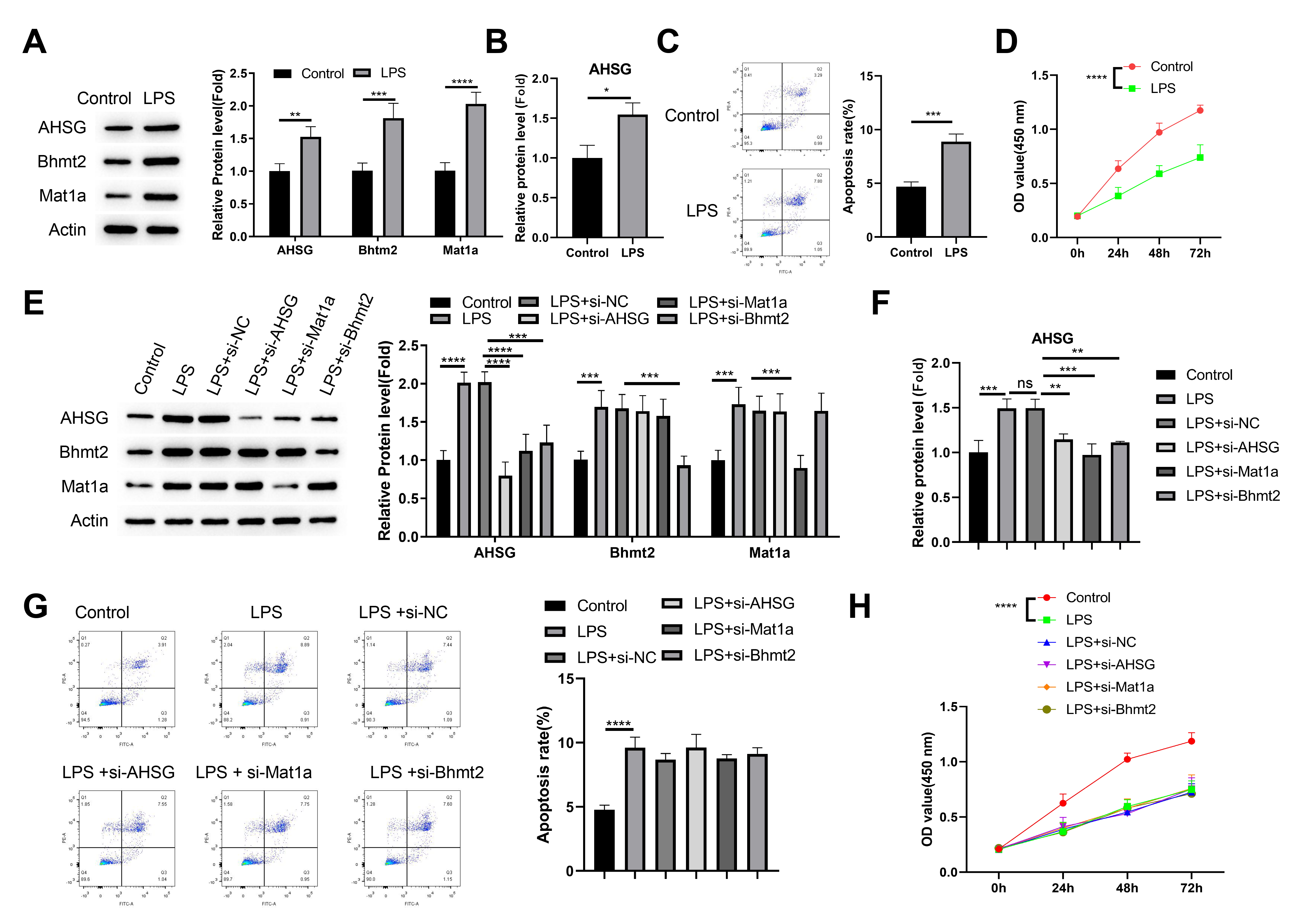


Actin

Membrane with ladder

Exposed signal


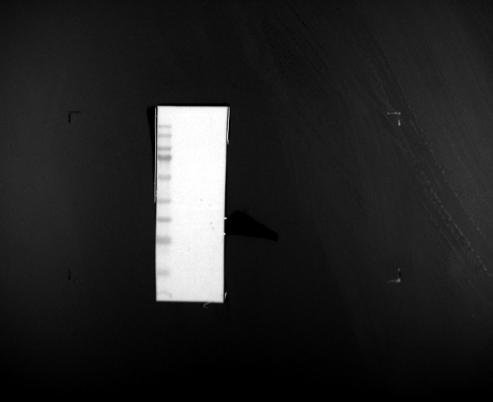

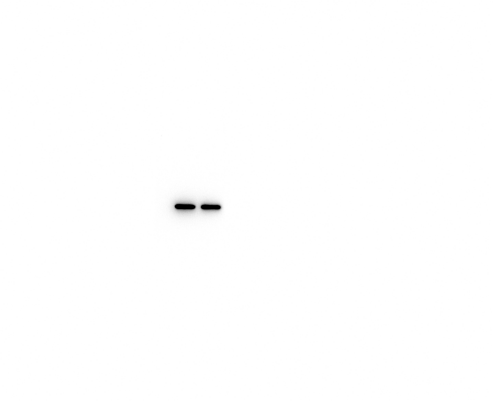

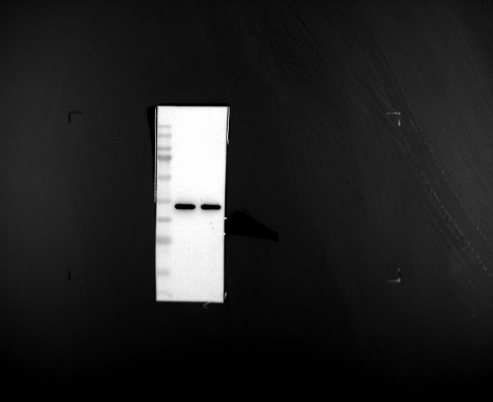


Molecular weight: 45 kDa

Merge

AHSG

Membrane with ladder

Exposed signal


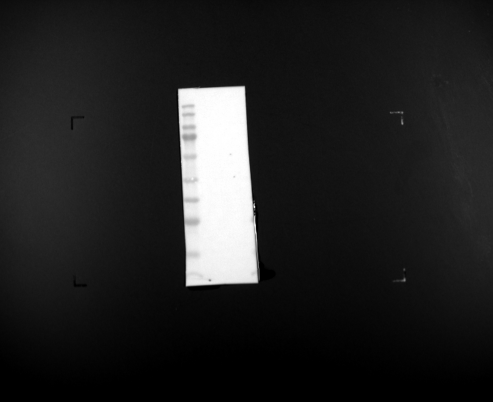

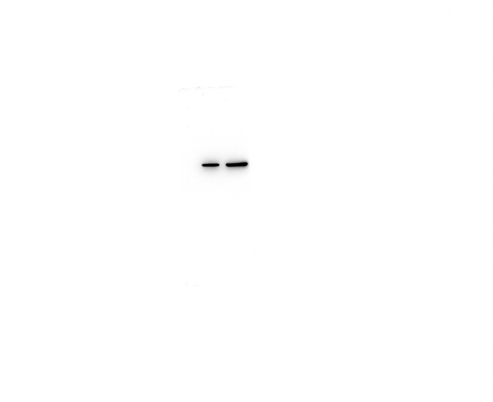

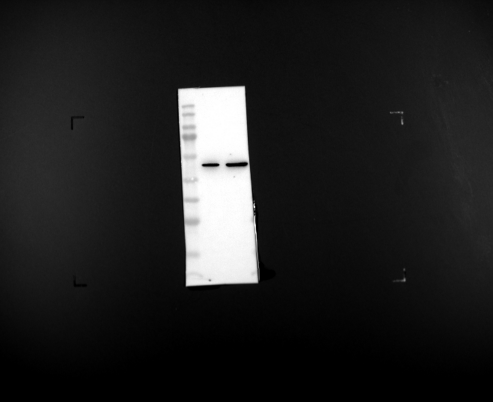


Molecular weight:55 kDa

Merge

Bhmt2

Membrane with ladder

Exposed signal


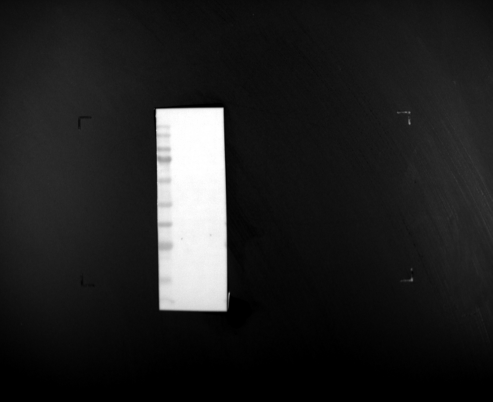

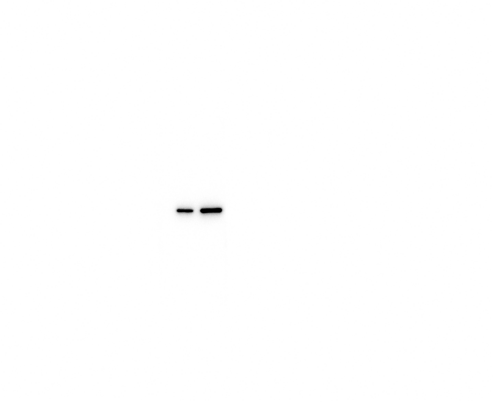

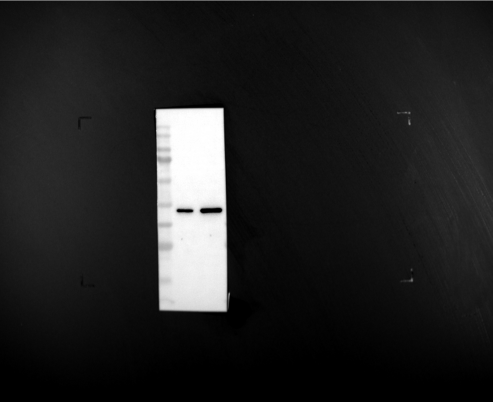


Molecular weight: 40-45 kDa

Merge

Mat1a

Membrane with ladder

Exposed signal


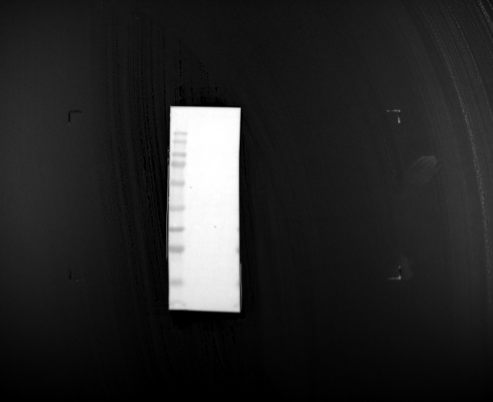

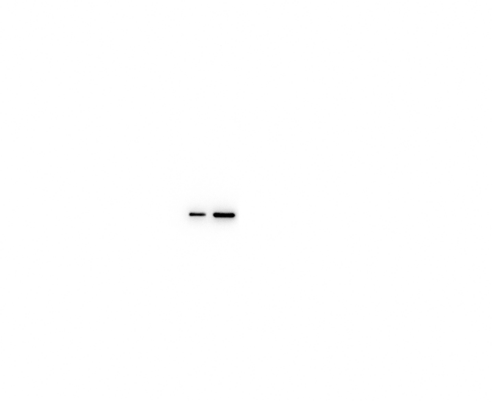

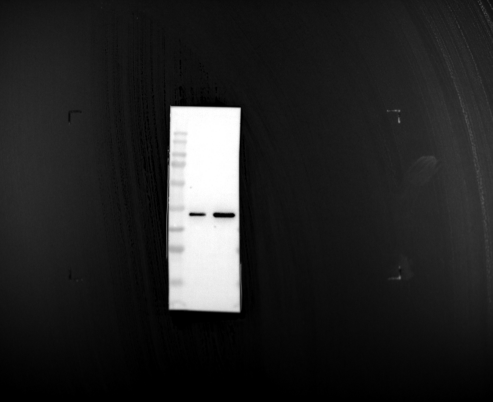


Molecular weight: 44 kDa

Merge

Figure 3E


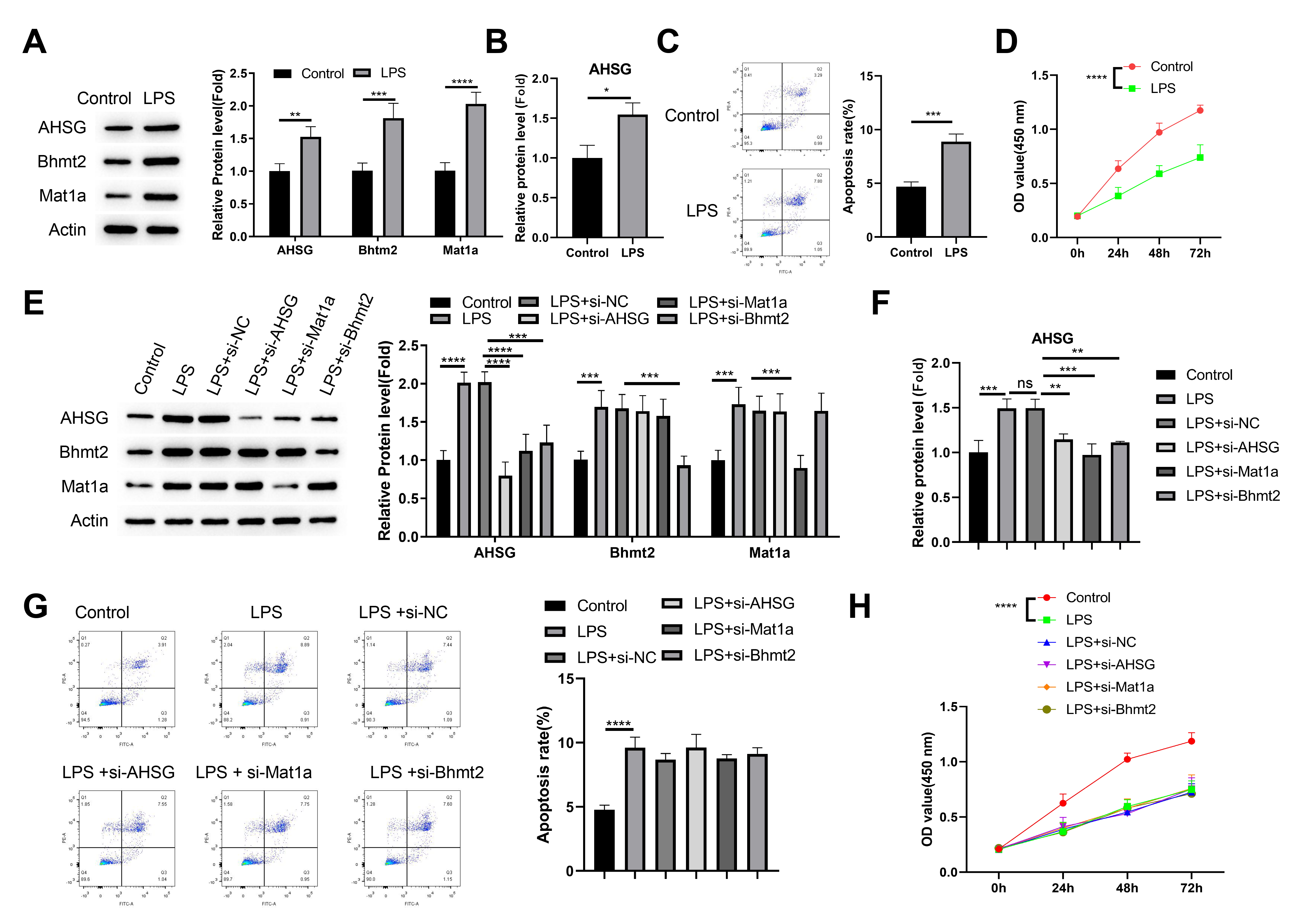


Actin

Membrane with ladder

Exposed signal


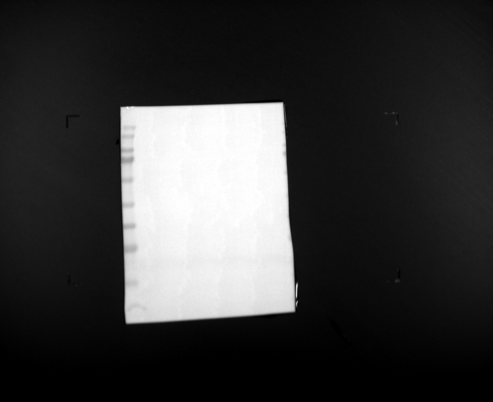

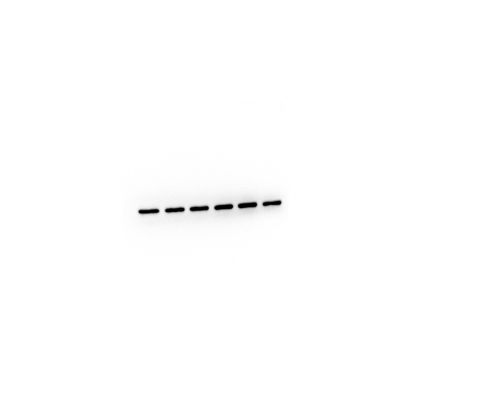

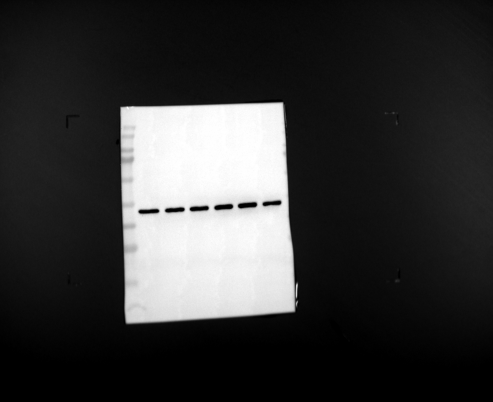


Molecular weight: 45 kDa

Merge

AHSG

Membrane with ladder

Exposed signal


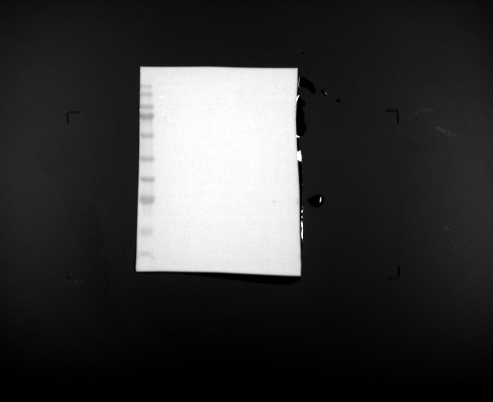

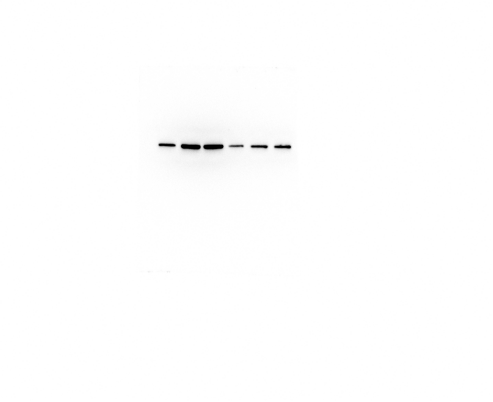

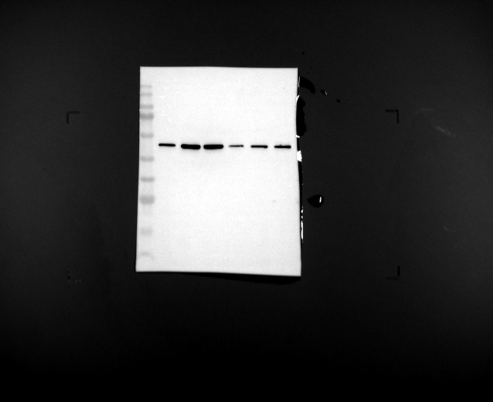


Molecular weight:55 kDa

Merge

Bhmt2

Membrane with ladder

Exposed signal


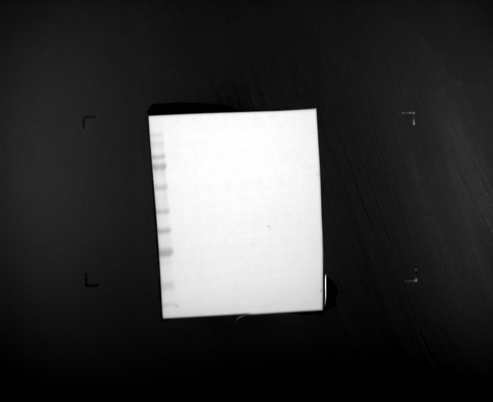

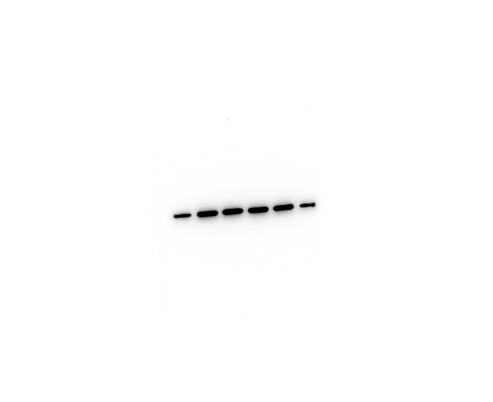

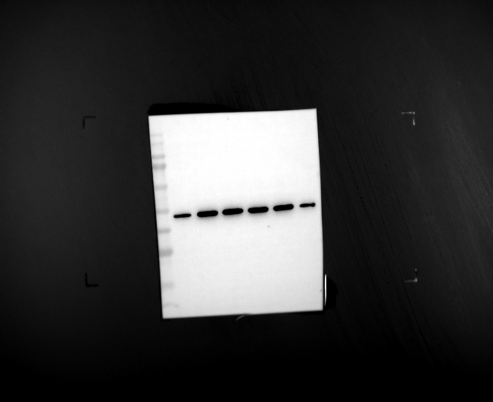


Molecular weight: 40-45 kDa

Merge

Mat1a

Membrane with ladder

Exposed signal


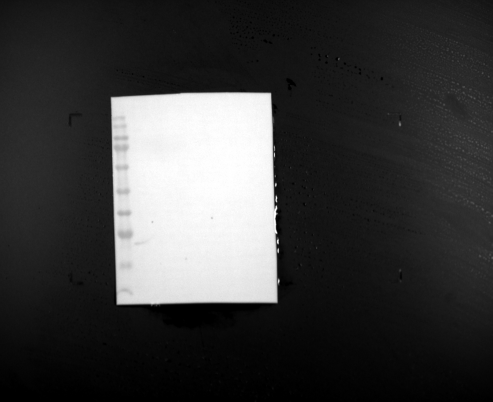

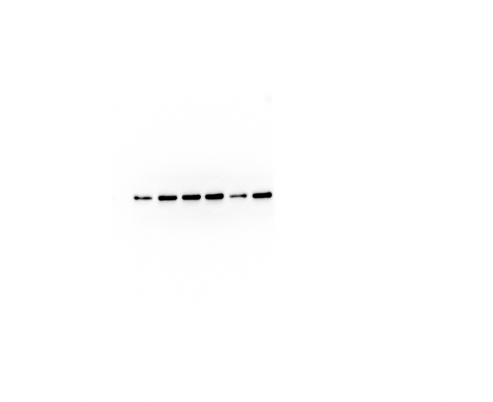

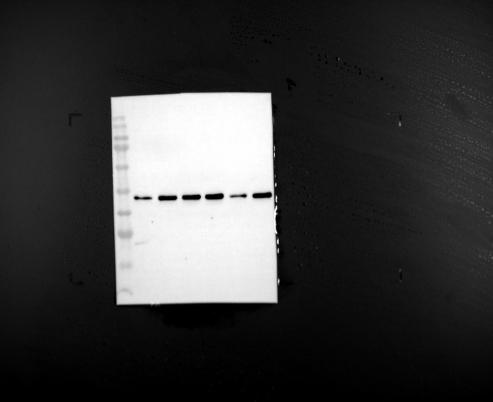


Molecular weight: 44 kDa

Merge

Figure 4F


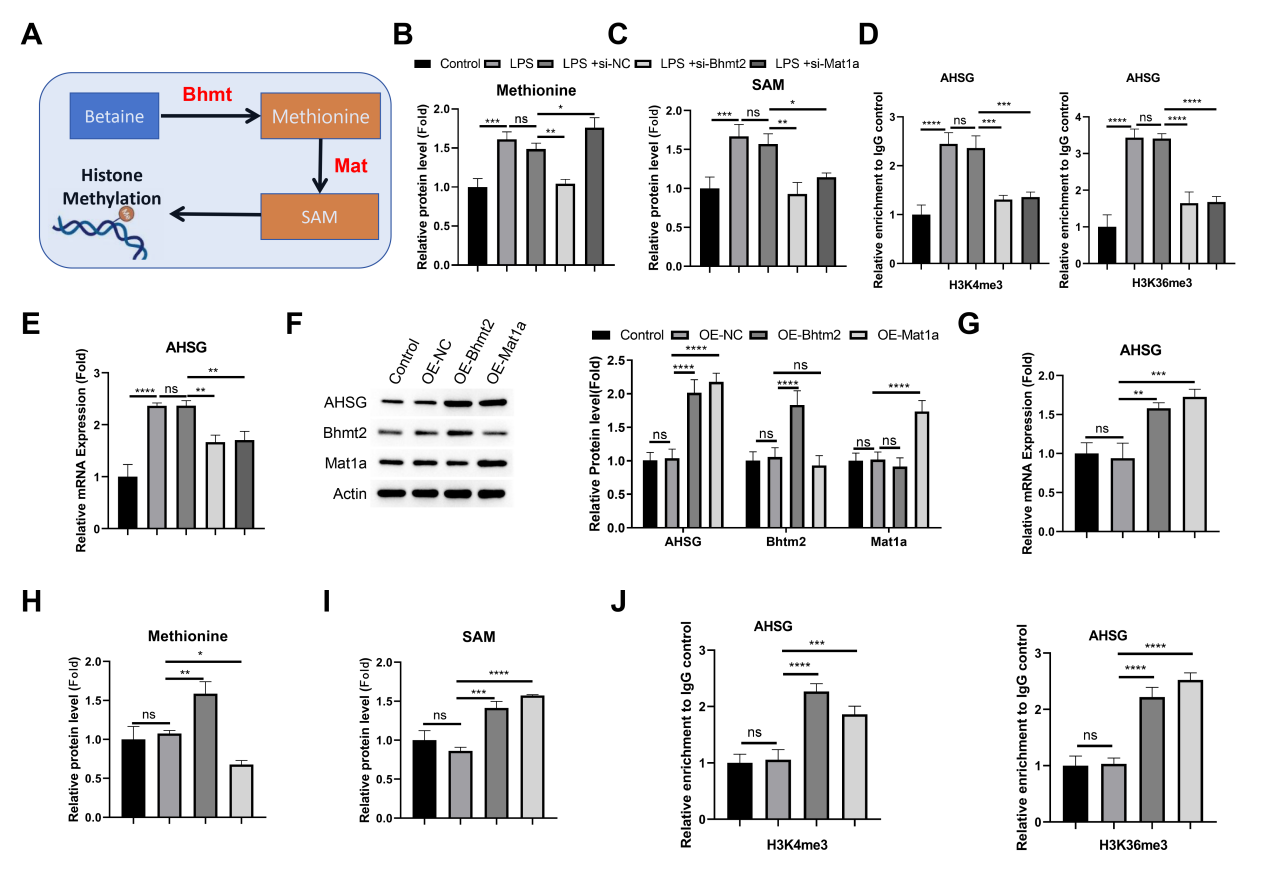


Actin

Membrane with ladder

Exposed signal


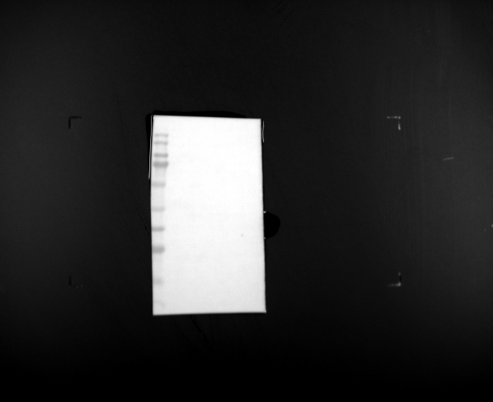

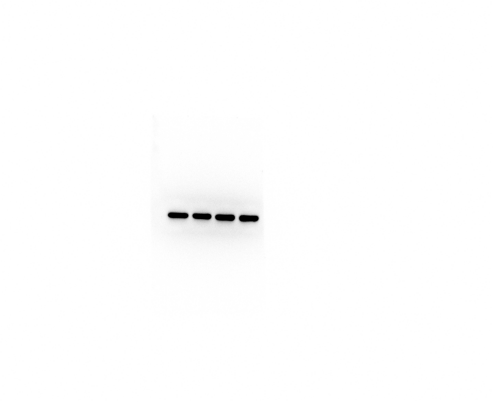

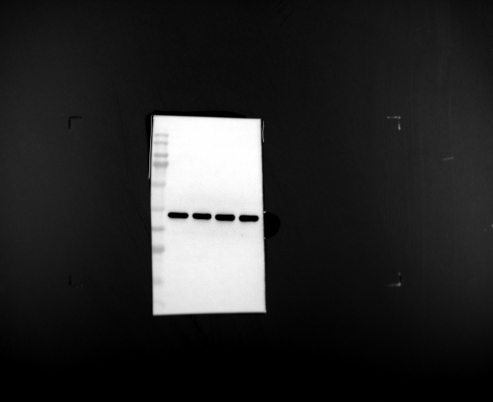


Molecular weight: 45 kDa

Merge

AHSG

Membrane with ladder

Exposed signal


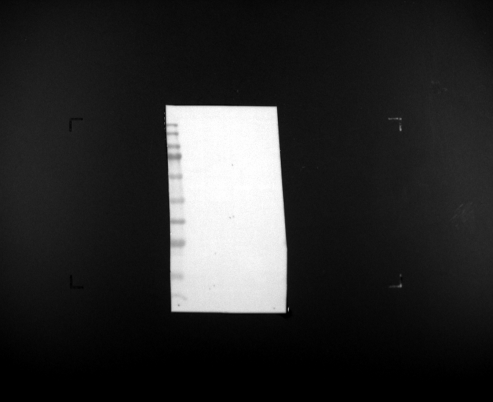

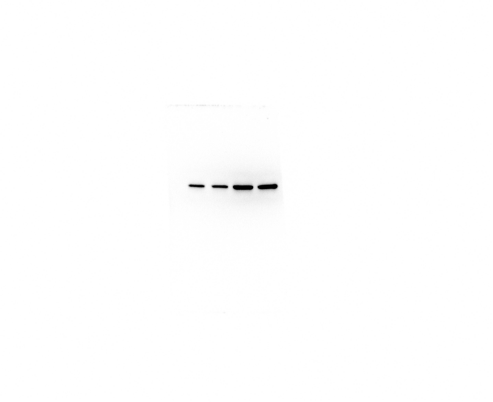

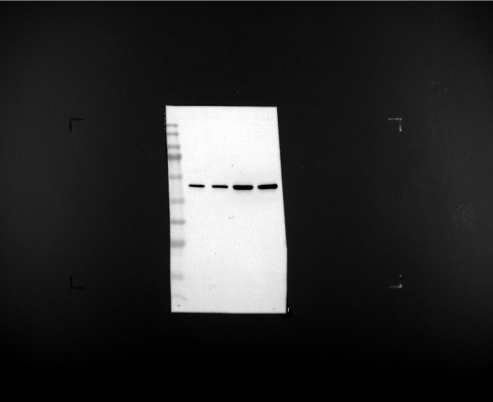


Molecular weight:55 kDa

Merge

Bhmt2

Membrane with ladder

Exposed signal


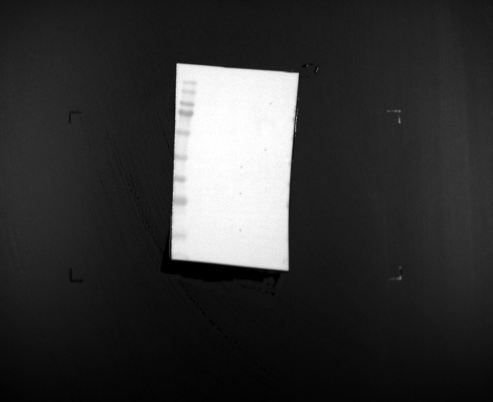

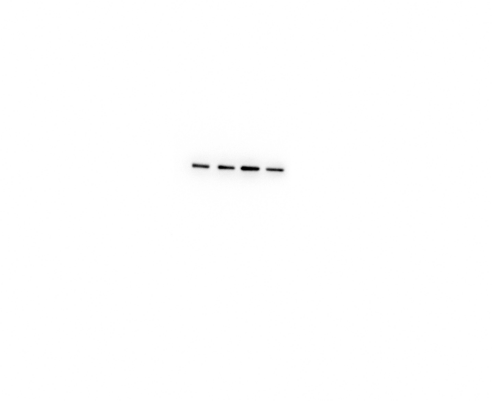

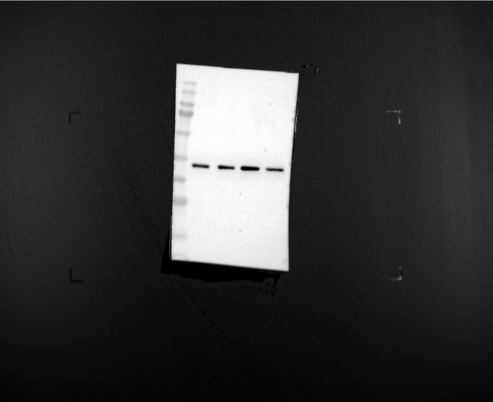


Molecular weight: 40-45 kDa

Merge

Mat1a

Membrane with ladder

Exposed signal


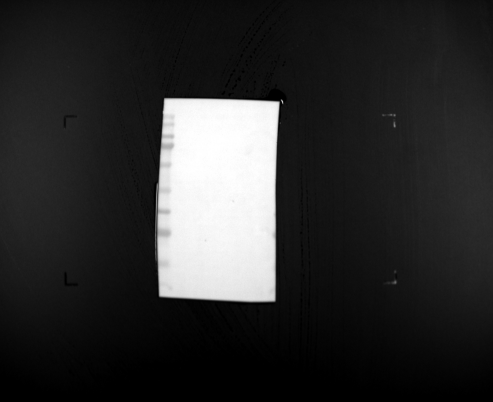

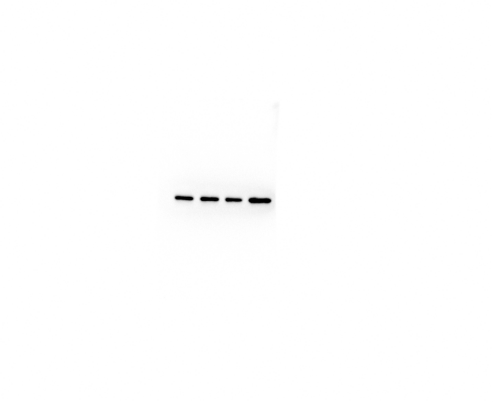

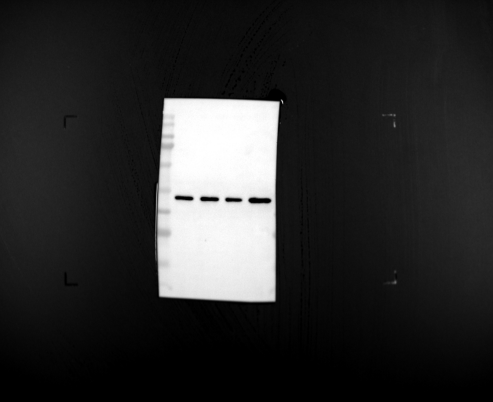


Molecular weight: 44 kDa

Merge

Figure 7A


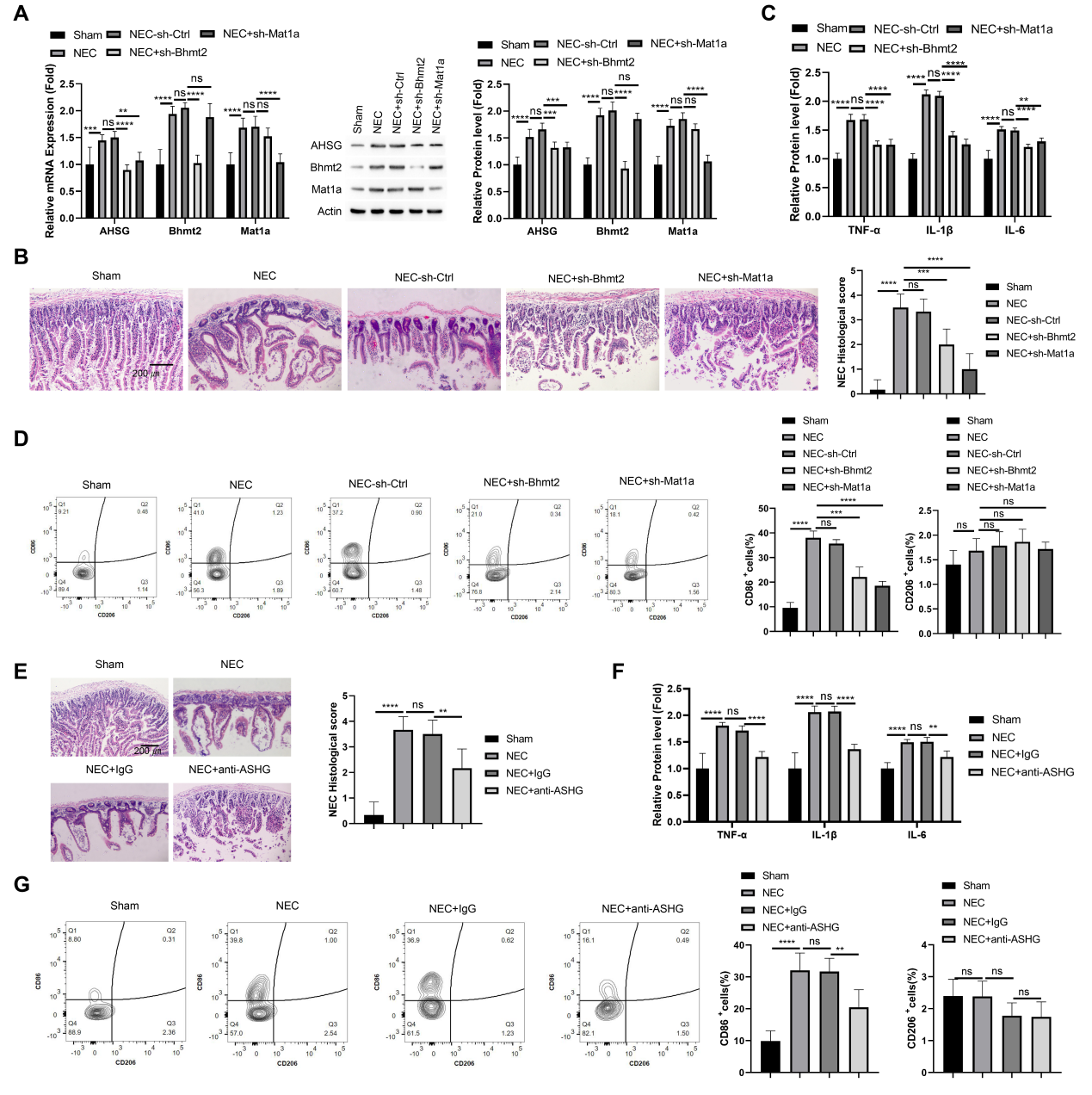


Actin

Membrane with ladder

Exposed signal


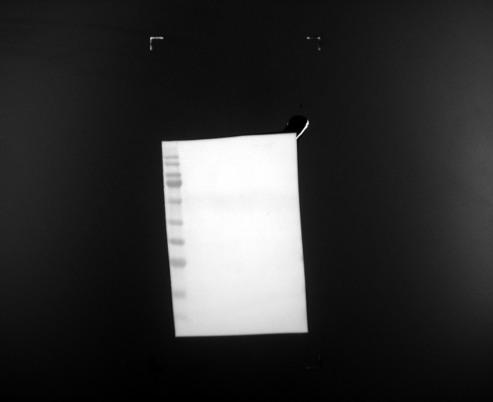

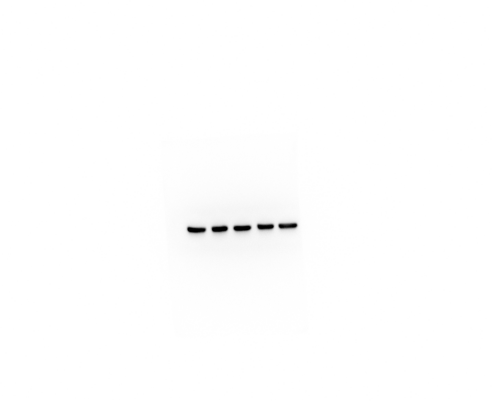

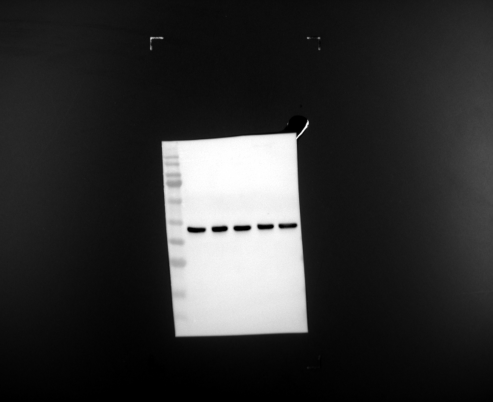


Molecular weight: 45 kDa

Merge

AHSG

Membrane with ladder

Exposed signal


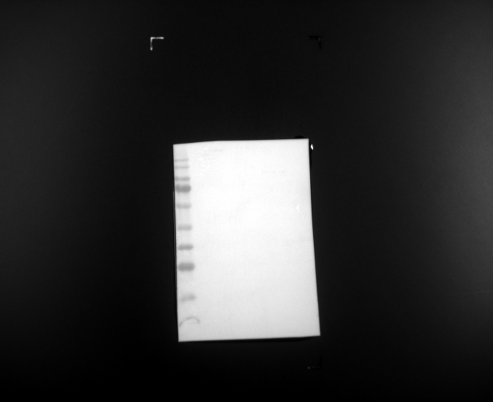

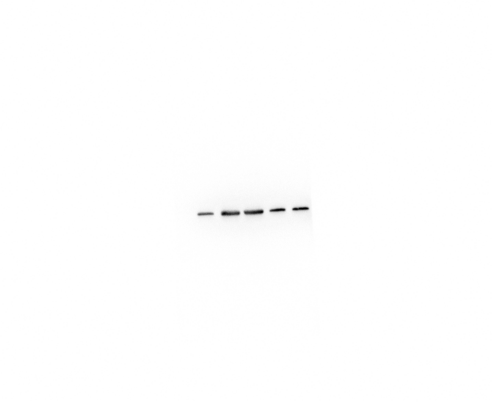

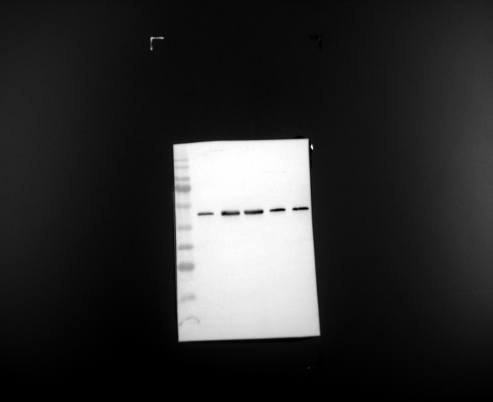


Molecular weight:55 kDa

Merge

Bhmt2

Membrane with ladder

Exposed signal


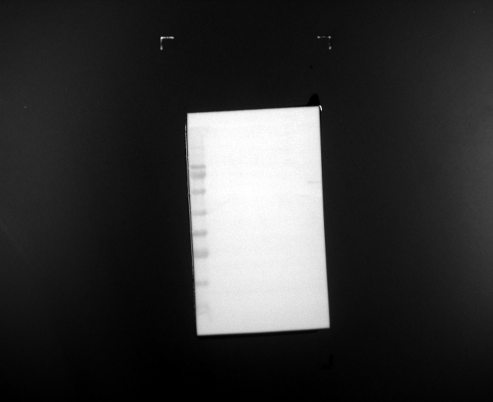

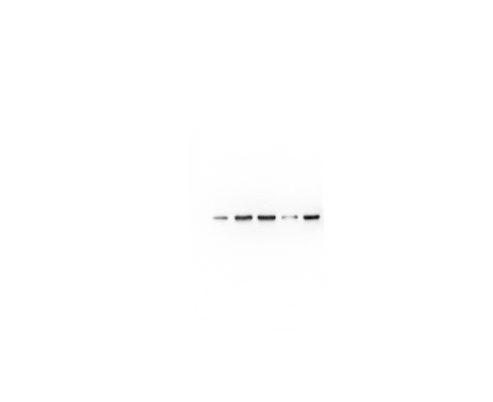

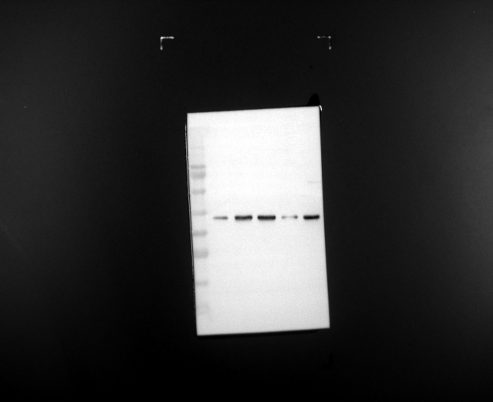


Molecular weight: 40-45 kDa

Merge

Mat1a

Membrane with ladder

Exposed signal


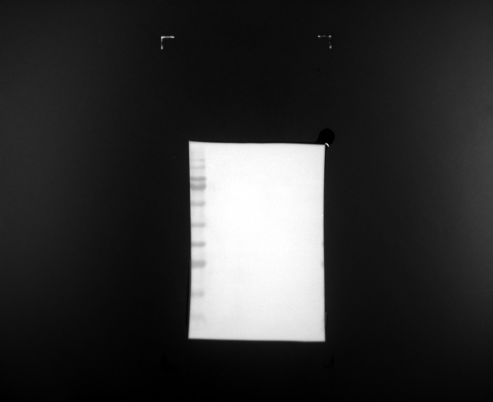

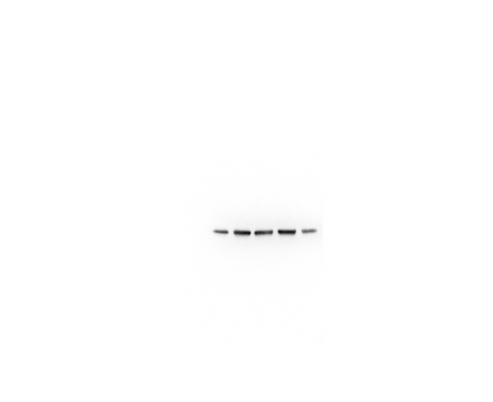

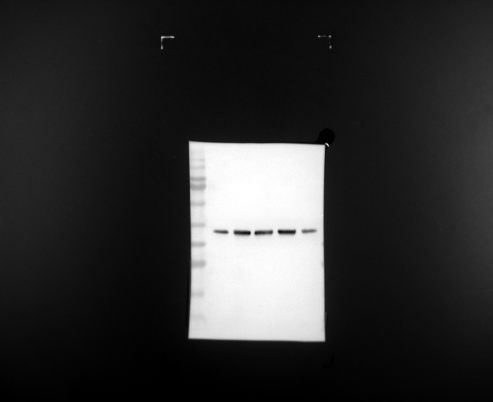


Molecular weight: 44 kDa

Merge
